# Artificial intelligence-based multi-modal multi-tasks analysis reveals tumor molecular heterogeneity, predicts preoperative lymph node metastasis and prognosis in papillary thyroid carcinoma: a retrospective study

**DOI:** 10.1097/JS9.0000000000001875

**Published:** 2024-07-11

**Authors:** Yunfang Yu, Wenhao Ouyang, Yunxi Huang, Hong Huang, Zehua Wang, Xueyuan Jia, Zhenjun Huang, Ruichong Lin, Yue Zhu, Yisitandaer yalikun, Langping Tan, Xi Li, Fei Zhao, Zhange Chen, Wenting Li, Jianwei Liao, Herui Yao, Miaoyun Long

**Affiliations:** a Guangdong Provincial Key Laboratory of Malignant Tumor Epigenetics and Gene Regulation, Department of Thyroid Surgery, Department of Medical Oncology, Breast Tumor Centre, Phase I Clinical Trial Centre, Sun Yat-sen Memorial Hospital, Sun Yat-sen University, Guangzhou, China; b Faculty of Medicine, Macau University of Science and Technology, Taipa, Macao, China; c Department of Experimental Research, The Affiliated Tumor Hospital of Guangxi Medical University, Xi Guang, China; d Guilin Medical University, Xi Guang, China; e Faculty of Innovation Engineering, Macau University of Science and Technology, Taipa, Macao, China; f School of Computer Engineering, Guangzhou Huali College, Guangzhou, China; g Burning Rock Biotech, Guangzhou, China; h Singleron Biotechnologies, Guangzhou, China; i Cellular and Molecular Diagnostics Center, Sun Yat-sen Memorial Hospital, Sun Yat-sen University, Guangzhou, China

**Keywords:** artificial intelligence, disease-free survival, lymph node metastases, molecular heterogeneity, multi-model analysis, papillary thyroid carcinoma

## Abstract

**Background:**

Papillary thyroid carcinoma (PTC) is the predominant form of thyroid cancer globally, especially when lymph node metastasis (LNM) occurs. Molecular heterogeneity, driven by genetic alterations and tumor microenvironment components, contributes to the complexity of PTC. Understanding these complexities is essential for precise risk stratification and therapeutic decisions.

**Methods:**

This study involved a comprehensive analysis of 521 patients with PTC from our hospital and 499 patients from The Cancer Genome Atlas (TCGA). The real-world cohort 1 comprised 256 patients with stage I–III PTC. Tissues from 252 patients were analyzed by DNA-based next-generation sequencing, and tissues from four patients were analyzed by single-cell RNA sequencing (scRNA-seq). Additionally, 586 PTC pathological sections were collected from TCGA, and 275 PTC pathological sections were collected from the real-world cohort 2. A deep-learning multi-modal model was developed using matched histopathology images, genomic, transcriptomic, and immune cell data to predict LNM and disease-free survival (DFS).

**Results:**

This study included a total of 1011 PTC patients, comprising 256 patients from cohort 1, 275 patients from cohort 2, and 499 patients from TCGA. In cohort 1, the authors categorized PTC into four molecular subtypes based on BRAF, RAS, RET, and other mutations. BRAF mutations were significantly associated with LNM and impacted DFS. ScRNA-seq identified distinct T-cell subtypes and reduced B-cell diversity in BRAF-mutated PTC with LNM. The study also explored cancer-associated fibroblasts and macrophages, highlighting their associations with LNM. The deep-learning model was trained using 405 pathology slides and RNA sequences from 328 PTC patients and validated with 181 slides and RNA sequences from 140 PTC patients in the TCGA cohort. It achieved high accuracy, with an area under the curve (AUC) of 0.86 in the training cohort, 0.84 in the validation cohort, and 0.83 in the real-world cohort 2. High-risk patients in the training cohort had significantly lower DFS rates (*P*<0.001). Model AUCs were 0.91 at 1 year, 0.93 at 3 years, and 0.87 at 5 years. In the validation cohort, high-risk patients also had lower DFS (*P*<0.001); the AUCs were 0.89, 0.87, and 0.80 at 1, 3, and 5 years. The authors utilized the GradCAM algorithm to generate heatmaps from pathology-based deep-learning models, which visually highlighted high-risk tumor areas in PTC patients. This enhanced clinicians’ understanding of the model’s predictions and improved diagnostic accuracy, especially in cases with lymph node metastasis.

**Conclusion:**

The artificial intelligence (AI)-based analysis uncovered vital insights into PTC molecular heterogeneity, emphasizing BRAF mutations’ impact. The integrated deep-learning model shows promise in predicting metastasis, offering valuable contributions to improved diagnostic and therapeutic strategies.

## Introduction

Highlights

- Molecular subtyping of cancer: The study categorizes papillary thyroid carcinoma (PTC) into four distinct molecular subtypes based on mutations in BRAF, RAS, RET, and other genes. Each molecular subtype is characterized by a unique tumor microenvironment (TME), contributing to the overall complexity of PTC.
- Association of BRAF mutations with lymph node metastasis: The research identifies a significant association between BRAF mutations and lymph node metastasis (LNM), impacting disease-free survival (DFS). This highlights the clinical relevance of specific genetic alterations in predicting the spread of PTC to lymph nodes, thereby influencing patient prognosis.
- Depth exploration of tumor microenvironment components: we employ scRNA-seq to reveal distinct T-cell subtypes and their roles in the TME. Notably, it uncovers reduced diversity in B cells within BRAF-mutated PTC with LNM. Additionally, the research explores the diversity of cancer-associated fibroblasts and macrophage subtypes, emphasizing their associations with LNM.
- Implications for clinical decision-making: To enhance diagnostic and prognostic accuracy, a novel deep-learning-based multi-modal model integrates diverse data sources, showcasing high accuracy in predicting preoperative LNM and risk stratification in PTC.

Papillary thyroid carcinoma (PTC) is the most common form of thyroid cancer, accounting for the majority of cases worldwide. Despite its relatively favorable prognosis, a subset of PTC patients experiences lymph node metastasis (LNM), which is associated with increased morbidity and mortality^1,2^. The presence or absence of PTC LNM substantially influences the choice of surgical procedures and treatment plans^3^. LNM is frequently influenced by a myriad of factors, and tumor heterogeneity stands out as a significant contributing element. In the realm of clinical practice, delving into or distinguishing heterogeneity in PTC proves challenging^4^. Hence, comprehending the molecular intricacies inherent in PTC and their correlation with LNM becomes paramount for enhancing risk stratification and providing guidance for informed therapeutic decisions.

In recent years, advances in high-throughput omics technologies have provided unprecedented opportunities to unravel the molecular landscape of PTC. Integrative multi-modal analysis, encompassing genomic, transcriptomic, and proteomic data, has emerged as a powerful approach to comprehensively characterize the genetic alterations and molecular pathways involved in PTC pathogenesis. Previous studies have identified several key genetic alterations in PTC, with BRAF mutations being the most prevalent, followed by RAS and RET mutations^5–7^. In particular, as the most common type of mutation, BRAF mutation is associated with MAPK pathway activation, which results in changing of the tumor microenvironment (TME) of PTC^8,9^.

Understanding the role of TME in PTC is crucial, as it significantly influences tumor progression and metastasis. This knowledge is key to unraveling the disease’s mechanisms and identifying therapeutic targets^10,11^. The complexity of PTC is further highlighted by recent advances in single-cell RNA sequencing (scRNA-seq). This technology has become pivotal in dissecting the intricate details of cell clusters and their gene functions within complex mixtures. It has been instrumental in revealing the depth of tumor heterogeneity in PTC, tracing the tumor’s origin, and understanding its malignant evolution^12^. However, despite these advances, the molecular heterogeneity of PTC, particularly in cases with LNM, remains an area requiring further clarification. Therefore, exploring the relationship between mutation types, TME heterogeneity, and their roles in promoting LNM in PTC is of paramount importance. Such research could shed light on the mechanisms contributing to molecular heterogeneity in both PTC and LNM, potentially leading to more targeted and effective treatment strategies.

Artificial intelligence (AI) algorithms have spearheaded a transformative era in the integration of multi-modal data, resulting in heightened precision in cancer prognosis predictions and the identification of numerous clinically beneficial biomarkers^13^. This study aims to explore the molecular heterogeneity characteristics and the intricate landscape of TME in PTC. Furthermore, a deep-learning multi-modal model was developed by incorporating matched histopathology slide images, genomic, transcriptomic, immune cells data to predict LNM and disease-free survival (DFS) in PTC patients, thereby facilitating more accurate clinical decision-making.

## Method and material

### STROCSS criteria statement

The work has been reported in line with the STROCSS criteria, Supplemental Digital Content 1, http://links.lww.com/JS9/D56, and this study’s design, conduct, and reporting adhere to established guidelines for observational studies^14^.

### Patients and study design

This study included a total of 1011 PTC patients, comprising 256 patients from real-world cohort 1, 275 patients from real-world cohort 2, and 499 patients from TCGA. The real-world cohort consisted of a total of 531 patients with stages I–III PTC (cohort 1 + cohort 2) consecutively diagnosed between January 2019 and August 31, 2021. Real-world cohort 1 comprised 256 patients. Tumor tissues from 252 patients with PTC were analyzed by DNA-based next-generation sequencing (NGS). Formalin-fixed paraffin-embedded (FFPE) tissues were prospectively collected after surgery. All corresponding archived hematoxylin and eosin-stained (H&E) tumor sections were prospectively collected and independently reevaluated by experienced histopathologists. Tumor tissues from four patients with PTC were analyzed by single-cell RNA sequencing (scRNA-seq). The validated PTC cohort data were obtained from the TCGA data portal (https://www.cancer.gov/tcga/), which contains whole-exome sequencing and transcriptome RNA sequencing gene expression profiles of 468 patients with PTC. In addition, 861 pathological sections were collected, containing 586 PTC pathological sections from TCGA, and 275 pathological sections from real-world cohort 2. (Supplementary Table 1, Supplemental Digital Content 2, http://links.lww.com/JS9/D57).

### DNA isolation and capture-based targeted DNA sequencing

DNA isolation and targeted sequencing were performed and certified according to the optimized protocols described previously. Briefly, tissue DNA was extracted from FFPE tumor tissues and DNA concentration was measured. For NGS library preparation, DNA was subjected to end repair, phosphorylation, and adaptor ligation. Fragments measuring 200–400 bp in size were selected by beads, followed by hybridization with capture probe panels consisting of 18 PTC-related genes (Supplementary Table 2, Supplemental Digital Content 2, http://links.lww.com/JS9/D57), hybrid selection with magnetic beads, and PCR amplification. The panel comprised 18 genes that are closely relevant to the pathogenesis and development of PTC. After the assessment of the QC of the fragments, indexed samples were deep-sequenced with pair-end reads. The sequence data were mapped to the human genome (hg19). More details were described in the Supplementary Methods, Supplemental Digital Content 2, http://links.lww.com/JS9/D57.

### Single-cell transcriptome sequencing and analysis

We performed scRNA-seq analysis on unselected viable cells from four fresh PTC samples to elucidate the comprehensive transcriptomic landscape and intercellular communication network. The fresh tissues were stored on ice after the surgery within 30 min. The four PTC tissues were made to a single-cell suspension in PBS, which were applied to the microfluidic chip and constructed a scRNA-seq library. After Primary analysis of raw read data, quality control, dimension-reduction and clustering, data from scRNA-seq were under generation and analysis, including differentially expressed genes (DEGs) analysis, cell type annotation, pathway enrichment analysis, etc. More details about scRNA-seq were described in the Supplementary Methods, Supplemental Digital Content 2, http://links.lww.com/JS9/D57.

### Training and validating the multi-modal multi-tasks models by deep learning

We built a multi-modal a high-throughput, understandable, weakly-supervised, and multi-modal deep-learning algorithm that is specifically devised for amalgamating whole-slide digitized histological images (WSIs) and molecular profile data in multi-tasks weakly-supervised learning. The multi-modal deep learning model uses the backbone of the pre-trained-ResNet50 with self-attention module to deal with the WSIs, and a concanative way to fusion the different omics data. In the initial stage, we initiated the process by utilizing the WSIs processing techniques from CLAM^15^, which enabled tissue segmentation and patching of each slide. This approach empowered us to enhance efficiency by retaining only the valid areas that would be cropped into multiple 256×256 RGB images without any overlap at the 20× equivalent pyramid level from all the identified tissue regions. Subsequently, we performed targeted feature extraction on each partitioned section using a pre-trained ResNet50^16^ model.

To integrate different feature in the training stage, we adopted a state-of-the-art attention gate model^17^, which can automatically extract the most efficient regions for DFS and LNM analysis from the WSIs. In the multi-modal deep-learning framework, each gigapixel WSI is partitioned into smaller regions and considered as a collection of patches with a corresponding slide-level label used for training. Furthermore, we synchronously embedded molecular and clinical feature with WSIs after passing through the attention gate by concatenating to enhance prediction accuracy. Finally, the final DFS risk scores or the LNM were predicted after one fully connected layers as the regressor or classifier.

In order to carry out prognostication for survival from patient-level survival data subject to right-censoring, for prognostication of survival from patient-level survival data subject to right-censoring, we employ the negative log-likelihood 
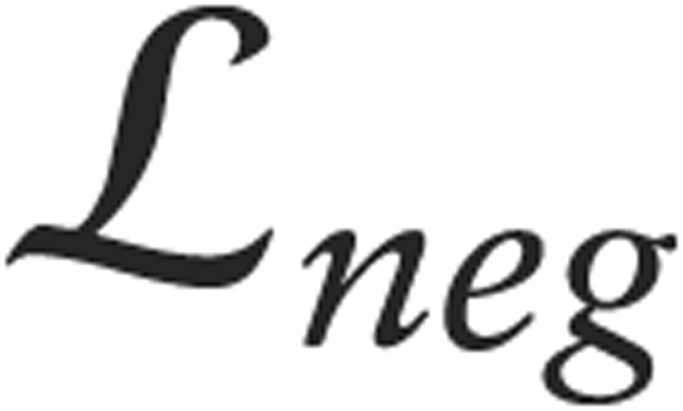
 as the loss function for survival prediction. Moreover, we make use of cross entropy (CE) loss 
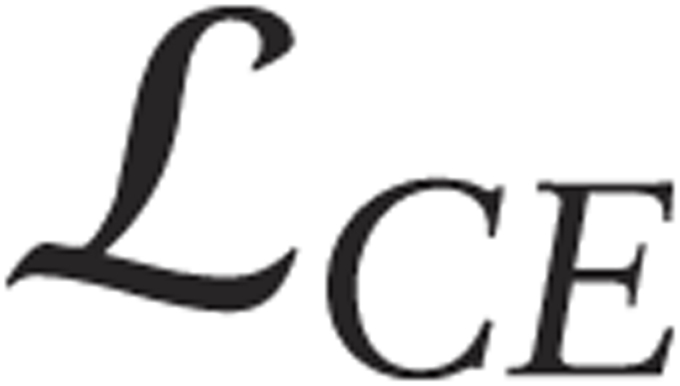
 as the loss function for LNM classification. Additionally, for DFS analysis, we utilize the Mean Squared Error (MSE) loss function 
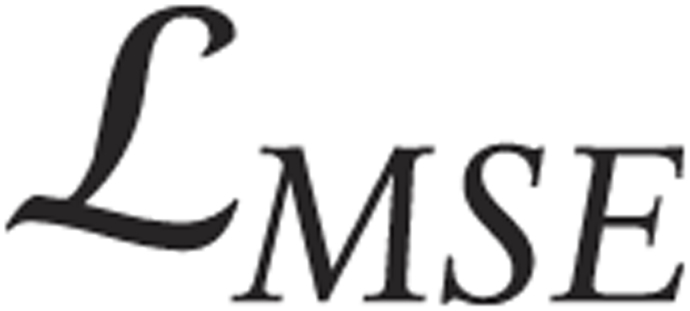
 to predict the time, aiming to achieve a close approximation to the actual time.

**Figure:**
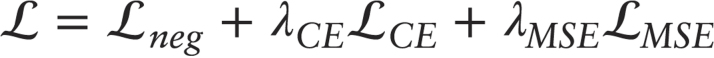


The combination of these two loss functions will be adjusted by tuning the values of $\lambda_{\mathrm{CE}}$ and $\lambda_{\mathrm{MSE}}$ .

### Implementation details

The training process was executed on both single and multi-GPU setups, utilizing the Adam optimizer with a learning rate of $1\times 10^{-4}\;$ and a weight decay of $1\times 10^{-5}$ . The training was conducted for a maximum of 20 epochs, with dropout applied at a probability of 0.25 to enhance the model’s generalization capability. Early stopping was implemented to prevent overfitting, halting the training process if the validation performance did not improve over a predefined number of epochs. The model training scripts were configured to run with specific GPU settings, ensuring optimal utilization of computational resources. This rigorous training procedure was designed to ensure that the model learned effectively from the data while maintaining a balance between underfitting and overfitting.

Since the cohort contains only WSIs without corresponding gene expression data, we employed a specific strategy to validate the model’s performance on this dataset. During validation, all model weights were frozen to prevent further updates and ensure that the model’s learned features remained unchanged. To accommodate the absence of gene data, we set the input gene values to zero. This approach allowed us to maintain the same number of parameters in the model, ensuring a consistent evaluation process without altering the model’s architecture or complexity. By freezing the weights and setting gene inputs to zero, we could accurately assess the model’s capability to generalize and perform solely based on the visual features extracted from the WSIs.

### Visualization and interpretation of the model

To comprehensively analyze the image areas and features influencing the network’s output, we employ advanced visualization techniques, enabling a deeper understanding of its underlying mechanisms. The generated heatmaps highlight regions of interest within the WSIs, allowing for a clear visual representation of the model’s attention distribution. This method, utilizing attention-based learning, automatically generated attention scores for all tissue regions from the slides, which were displayed as attention heatmaps. These heatmaps assist clinicians in pinpointing critical tumor regions. By applying softmax, the attention scores were transformed into percentages and then represented as RGB color-coded heatmaps. Regions with higher attention scores were highlighted as potential diagnostic tumor tissue, while regions with lower scores were deemed normal tissue. The CLAM framework streamlined the prediction process by eliminating the need for WSI annotation, thus identifying significant regions more efficiently.

### Statistical analyses

Continuous and categorical variables are expressed as the mean ± standard deviation and numbers (percentages), respectively. The Kruskal–Wallis test and the chi-squared test were used where appropriate. Gene co-expression networks were constructed date of evaluation and the date of recurrence or date of last known status. Survival curves were plotted with the Kaplan–Meier method with log-rank statistics. Statistical significance was defined as two-sided values of *P* less than 0.05. The performance evaluation of these predictions was carried out by examining the receiver operating characteristic (ROC) curves for sensitivity and specificity, with the area under the ROC curve serving as the performance metric. Statistical analyses were conducted with R software version 4.1.0 (http://www.r-project.org). The deep-learning model was constructed using China’s Tianhe-2 supercomputer platform with PyTorch (version 1.10.1) and Python (version 3.5).

## Results

### Characteristics of papillary thyroid carcinoma patients

**Figure 1:**
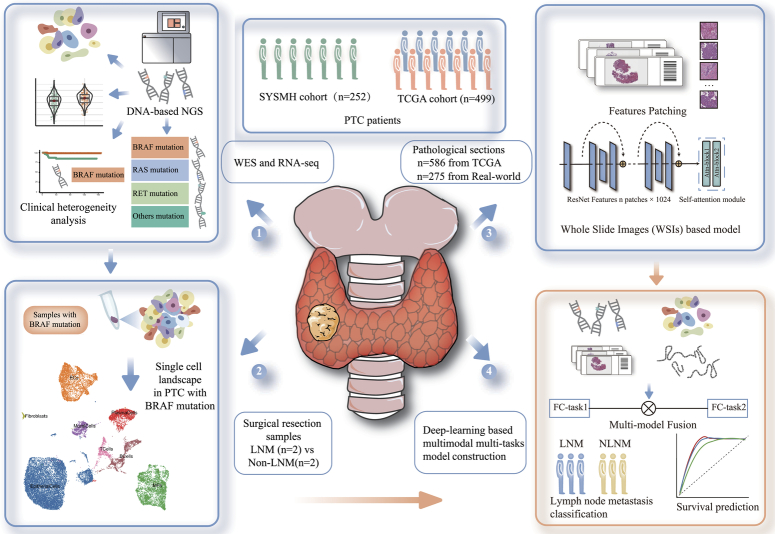
The overall flowchart of this study. Patient cohort selection: Include 1011 patients diagnosed with PTC. Divide patients into three cohorts: real-word cohort 1 (256 patients), real-word cohort 2 (275 patients) and TCGA cohort (499 patients). Subset the cohort 1 with consented four patients for scRNA-seq analysis. (cohort 1: Sun Yat-sen University of Sun Yat-sen University). LNM, lymph node metastasis; NLNM, non-lymph node metastasis; PTC, papillary thyroid carcinoma; TCGA, The Cancer Genome Atlas.

A total of 1011 patients diagnosed with PTC were included in this study, comprising 256 patients from cohort 1, 275 patients from cohort 2, and 499 patients from TCGA. Within cohort 1, tumor tissues from 252 patients with PTC were analyzed by DNA-based next-generation sequencing (NGS). Additionally, four consented patients [two with lymph node metastasis (LNM) and two without lymph node metastasis (NLNM)] underwent single-cell RNA sequencing (scRNA-seq) analysis. The remaining 252 patients in the cohort 1 [mean (SD) age, 42.1 (11.1) years; 188 (75%) female; 198 (79%) in stage I]. Among these 252 patients, 129 [51%] patients were classified as N stage. In the TCGA cohort [mean (SD) age, 47.3 (15.8) years; 365 (73%) female; 282 (57%) in stage I], 227 [45%] patients were classified as N stage. The most prevalent mutation observed in PTCs was BRAF, with mutation rates of 84% (212/252) and 60% (297/499) in the cohort 1 and TCGA cohorts, respectively. The overall design of this study was illustrated in Figure 1 and an overview of the general characteristics of the cohorts was shown in Supplementary Table 1, Supplemental Digital Content 2, http://links.lww.com/JS9/D57.

### Genetic alteration profiles and molecular subgroup characteristics in papillary thyroid carcinoma patients

**Figure 2:**
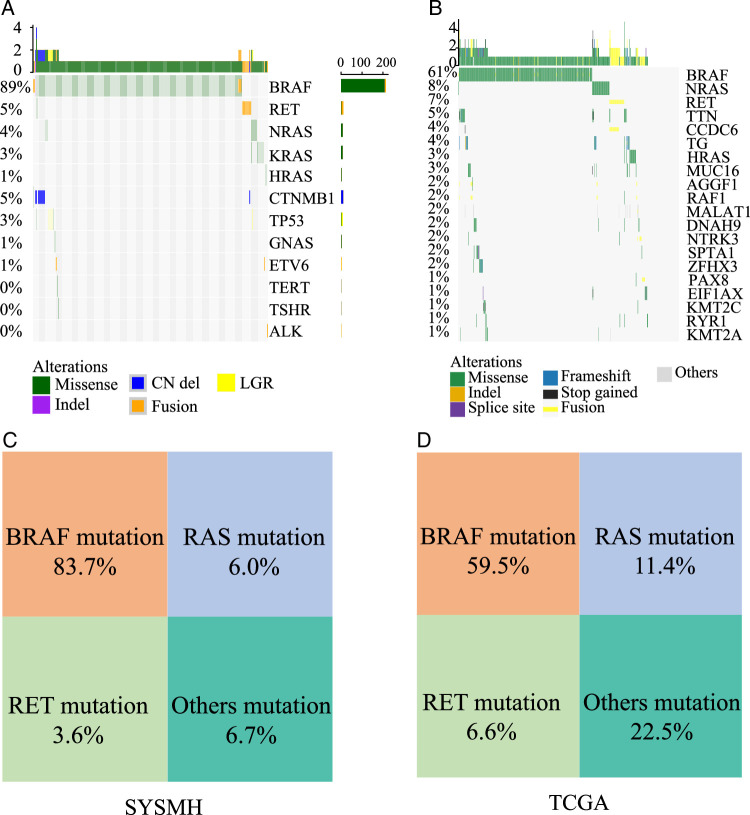
Genetic alteration profiles and molecular subgroup characteristics of PTC patients in cohort 1 and TCGA cohorts. (A) Clinicopathological characteristics and genetic alteration profiles of cohort 1. (B) Clinicopathological characteristics and genetic alteration profiles of cohort 1 TCGA cohorts. (C) molecular subgroups based on mutation status in cohort 1. (D) molecular subgroups based on mutation status in TCGA cohort. TCGA, The Cancer Genome Atlas.

The analysis of genetic alteration profiles in cohort 1 revealed that BRAF mutations were the most frequent mutation type in PTC, with a mutation rate of 89%. Similarly, in the TCGA cohort, BRAF mutations were the most common mutation gene type in PTC, accounting for 61% of the mutation ratio (Fig. 2A-B). In the cohort 1, the RAS gene, including HRAS, NRAS, and KRAS, was the second most common mutation in PTC patients, observed in 6.0% of cases (15/252). RET mutations ranked third, with a likelihood of 3.6% (9/252). In the TCGA cohort, the frequencies of BRAF, RAS, and RET gene mutations were 59.5% (297/499), 11.4% (57/499), and 6.6% (33/499), respectively. Collectively, these mutations (BRAF, RAS, RET) accounted for 93.2% of cohort 1 and 77.6% of the TCGA cohort, with BRAF, RAS, and RET mutations being mutually exclusive (Fig. 2C-D).

Based on these findings, PTC patients were reclassified into four molecular subgroups, considering BRAF, RAS, RET, and other molecular mutations. Among the four molecular subgroups in the cohort 1 and TCGA cohorts, RET mutations were associated with younger ages at diagnosis (cohort 1, Kruskal–Wallis test *P* = 0.062; TCGA, Kruskal–Wallis test *P* = 0.00034) Supplementary Figure S1A, C Supplemental Digital Content 2, http://links.lww.com/JS9/D57. PTCs driven by RAS and RET mutations exhibited larger tumor sizes compared to those driven by BRAF and other mutations(cohort 1, Kruskal–Wallis test *P* = 0.17) Supplementary Figure S1B, Supplemental Digital Content 2, http://links.lww.com/JS9/D57. Furthermore, patients with RAS mutations had a higher disease-free survival compared to patients with other mutations (*P*<0.05) Supplementary Figure S1D, Supplemental Digital Content 2, http://links.lww.com/JS9/D57. The rates of thyroid peroxidase positivity were higher in RET- and RAS-driven PTCs than in BRAF-driven PTCs in cohort 1 Supplementary Figure S1E, Supplemental Digital Content 2, http://links.lww.com/JS9/D57.

### Pathway analysis, immune checkpoint, and immune cell expression in papillary thyroid carcinoma molecular subgroups

**Figure 3:**
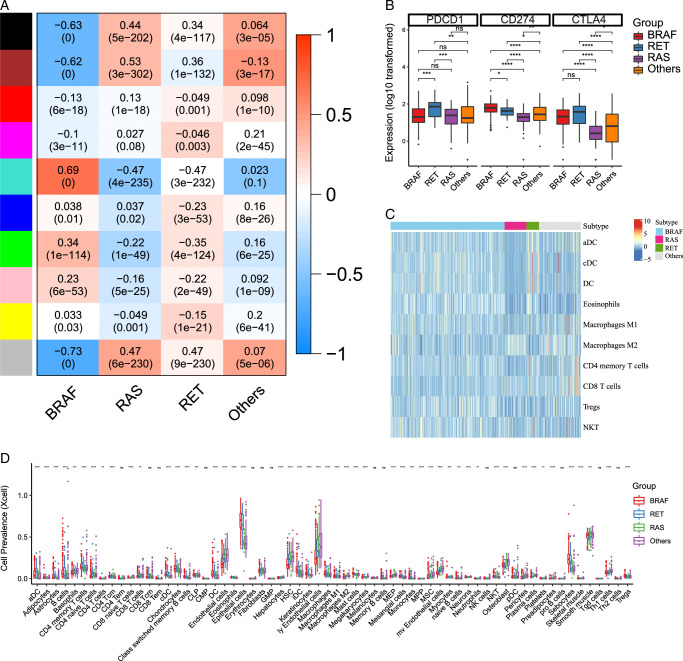
Common mutation-associated immune microenvironment in papillary thyroid carcinoma (PTC). (A) Heatmap shows the (weighted gene co-expression network analysis) of BRAF- RAS RET and others-driven PTCs. Top 10 enrichment results for turquoise, gray and black modules in PTC. (B) Analyzing the expression of PDCD1, CD274, and CTLA4 in different molecular subgroups in BRAF-, RET-, RAS and others-driven tumors. *<0.05 (C) Heatmap shows the expression of top 10 immune cells. The associated gene expression value, with red indicating high expression and blue indicating low expression. (D) Assessing immune cell infiltration obtained via Xcell algorithm in BRAF-, RET-, RAS and others-driven tumors. *<0.05.

To identify key pathways in PTC, we utilized weighted gene co-expression network analysis (WGCNA) to examine distinct modules within the four molecular subgroups. Based on gene similarities, we clustered 1000 genes into 10 separate modules (Fig. 3A).

Among the four molecular subgroups of PTC, we observed variations in immune checkpoint expressions. RAS-driven PTCs displayed significantly lower PD-1 expression compared to RET-driven PTCs, and significantly lower PD-L1 expression compared to both RET- and BRAF-driven PTCs (Fig. 3B). In contrast, BRAF-driven PTCs exhibited a significant increase in tumor-infiltrating Tregs (*P*<0.005). Additionally, BRAF-driven PTCs showed higher levels of NK and T-cell infiltration compared to RAS-driven PTCs. Using the xCell algorithm, we assessed the relative cell prevalence of 10 immune cells, revealing an anti-inflammatory tumor state for RAS-driven PTCs. These RAS-driven PTCs were associated with reduced levels of pro-inflammatory mediators, including dendritic cells (DCs), eosinophils, M1 macrophages, B cells, and CD4 memory T cells, along with increased levels of anti-inflammatory M2 macrophages (*P*<0.05) (Fig. 3C-D).

### Molecular heterogeneity, lymph node metastasis, and survival in papillary thyroid carcinoma

**Figure 4:**
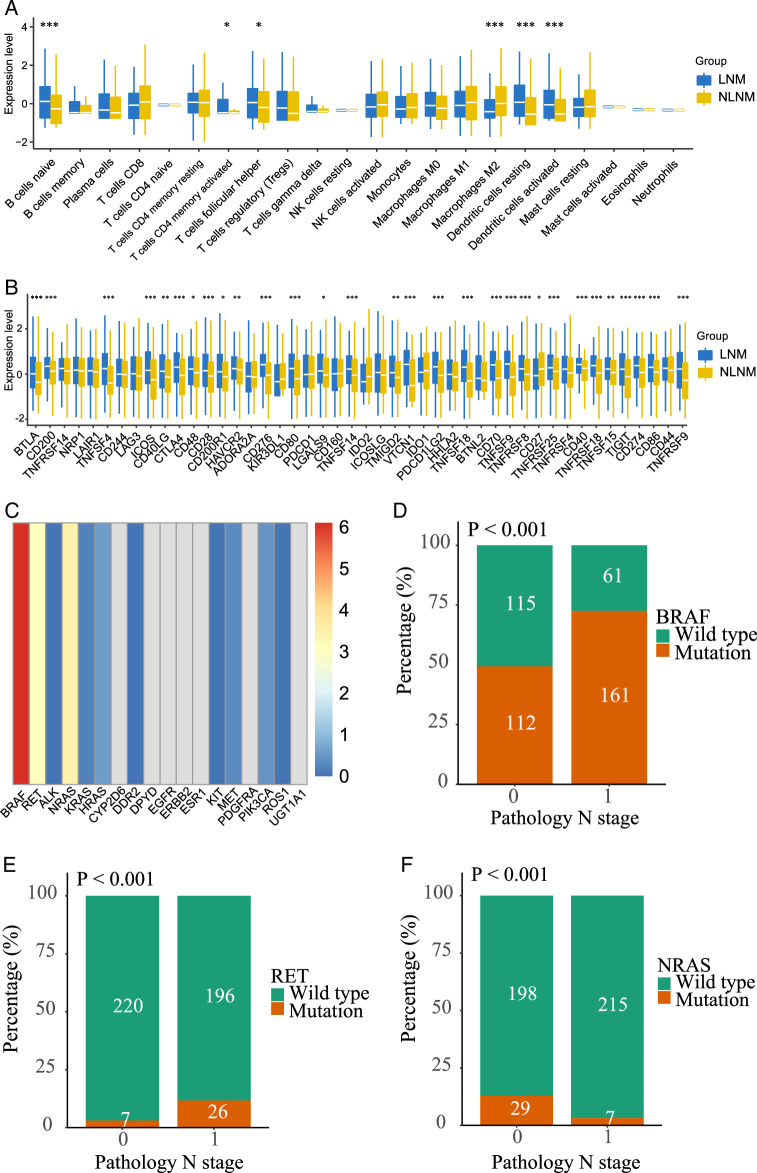
LNM and NLNM associated immune microenvironment in papillary thyroid cancer. (A) Changes of immune cells in lymph node metastases and non-lymph node metastases. (B) Changes of immune checkpoints in lymph node metastases and non-lymph node metastases. (C) Correlation analysis of 18 genes and lymph node metastasis and BRAF is the most relevant. (D) Metastasis ratio in patients with BRAF mutations (49.3% vs 72.5% *P*<0.001). (E) Metastasis ratio in patients with RET mutations (3.0% vs 11.7% *P*<0.001). (F) Metastasis ratio in patients with NRAS mutations (12.8% vs 3.2% *P*<0.001). LNM, lymph node metastasis; NLNM, non-lymph node metastasis**-**associated.

The molecular heterogeneity of PTC extends to the diversity of immune cell infiltration. In PTC patients with LNM, significantly higher levels of infiltration were observed for naive B cells, plasma cells, CD4 memory-activated T cells, follicular helper T cells, and dendritic cells (*P*<0.05) (Fig. 4A). Furthermore, PTC patients with LNM exhibited elevated expression of immune checkpoints such as PD-L1 and CTLA4 (*P*<0.05) (Fig. 4B). In cohort 1, BRAF mutations were found to be the most prevalent mutation type associated with LNM (Fig. 4C). The majority of patients with lymph node metastases had BRAF mutations (72.5% vs. 28.5%, *P*<0.001) (Fig. 4D). However, RET and RAS mutations were extremely rare in patients with LNM (*P*<0.001) (Fig. 4E-F).

Furthermore, TNM staging, a widely used model for evaluating PTC treatment strategies, was assessed. In the pre-categorized cohort (all patients), nodal-negative (N0) PTCs demonstrated significantly better DFS compared to patients with lymph node involvement (*P* = 0.018) Supplementary Figure S2A, Supplemental Digital Content 2, http://links.lww.com/JS9/D57. Patients were further stratified into three molecular subgroups: BRAF, RAS, and RET. Notably, following categorization by mutation status, the two primary N0 groups showed distinct DFS advantages. N0 patients with BRAF and RAS mutation-driven PTC had significantly better DFS than N1 patients (*P* = 0.0036, *P* = 0.012) Supplementary Figure S2B-C, Supplemental Digital Content 2, http://links.lww.com/JS9/D57. In contrast, no significant difference in DFS was observed between N1 and N0 patients with RET-mutation-driven PTC Supplementary Figure S2D, Supplemental Digital Content 2, http://links.lww.com/JS9/D57 Overall, the results suggest that N0 PTCs driven by BRAF and RAS mutations significantly impact DFS status, and the BRAF mutation is most strongly associated with LNM, indicating its connection to the immune microenvironment. However, no survival difference was observed between patients with non-BRAF-mutated lymph nodes and those without nodal metastases.

### Cellular composition heterogeneity in BRAF-Mutated papillary thyroid carcinoma with lymph node metastasis

**Figure 5:**
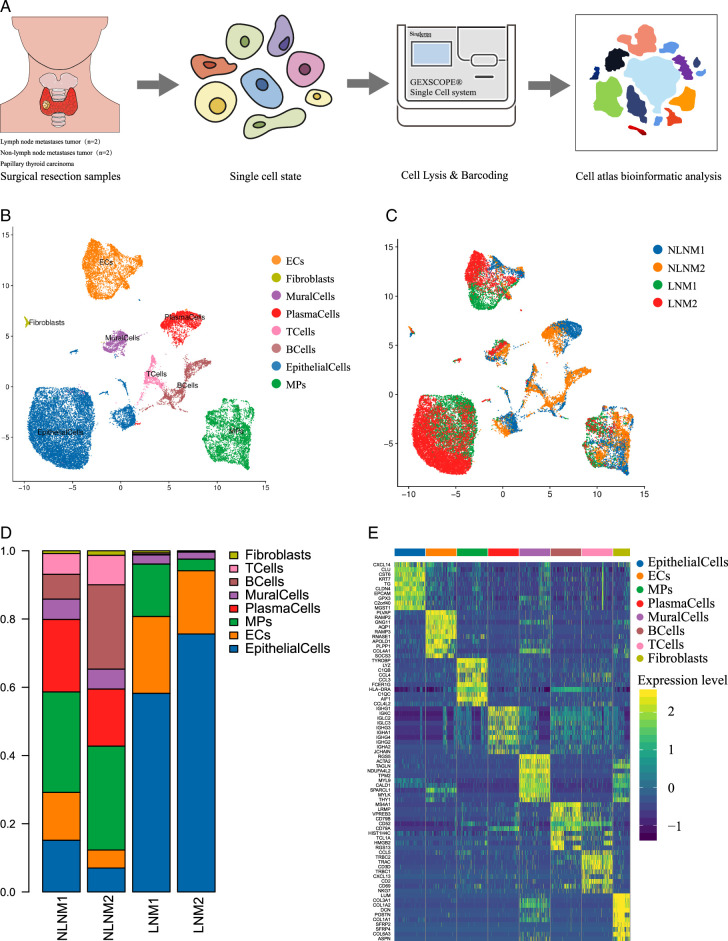
Intratumor heterogeneity among papillary thyroid cancer (PTC) histologic patterns. (A) Schematic diagram of scRNA-seq analysis workflow. Obtain surgical resection samples from BRAF-mutated PTC patients with and without LNM and performing single-cell RNA sequencing (scRNA-seq) on the samples. PTC and adjacent tissues were dissociated into single cells, sorted by FACS, and sequenced using GEXSCOPE® single-cell platform. (B) UMAP plots for the cell type identification of 27 351 high-quality single cells. (C) UMAP plots for the cell type identification in lymph node metastases and non-lymph node metastases. (D) Heatmap showing marker genes for 8 distinct cell types. (E) Heatmap showing 8 distinct cell types proportional change in lymph node metastases and non-lymph node metastases. LNM, lymph node metastasis; NLNM, non-lymph node metastasis.

To gain further insights into the landscape of LNM in BRAF-mutated PTC, we obtained surgical resection samples from four consented patients (two with LNM and two NLNM) in cohort 1. These samples underwent genomics scRNA-seq, resulting in a total of 27 040 single cells that were sequenced and subjected to further analysis to construct the cell atlas. Single-cell gene expression was normalized, and principal component analysis was employed for dimensionality reduction, followed by t-distributed stochastic neighbor embedding (tSNE) for clustering (Fig. 5A-B).

Based on the analysis of copy number variations (CNVs), we identified eight distinct cell types, including T cells, B cells, plasma cells, mononuclear phagocytes, fibroblasts, mural cells, endothelial cells, and epithelial cells, using reported marker genes. Notably, we discovered that the proportions of epithelial cells (which may vary in tumor cell invasion degree), endothelial cells, mononuclear phagocytes, plasma cells, B cells, and T cells exhibited the most significant differences between LNM groups and NLNM groups (Fig. 5C). Furthermore, the marker genes confirmed the accuracy of cell identity (Fig. 5D). Consequently, our findings suggest that the higher rate of lymph node metastasis in BRAF-mutated PTC may be attributed to cellular composition heterogeneity.

### Distinct T-cell subtypes and roles in PTC tumor microenvironment with lymph node metastasis

**Figure 6:**
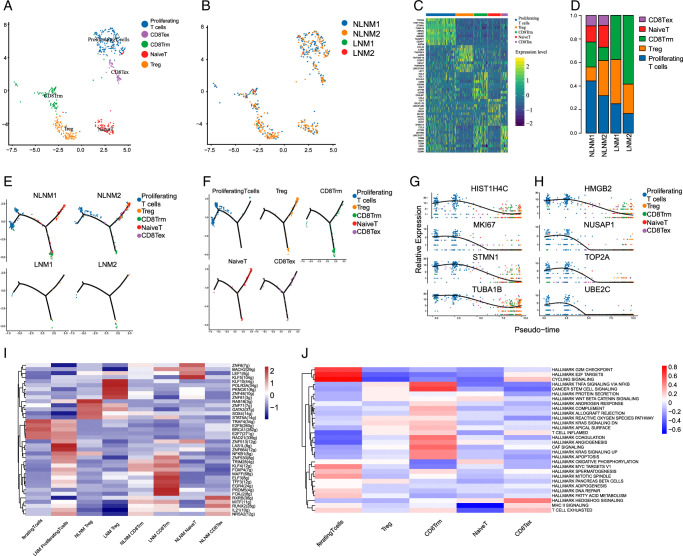
Distinct T-cell subtypes and roles in papillary thyroid cancer tumor microenvironment with LNM. (A) UMAP plots for the cell type identification of 6 different T-cell types based on marker genes. (B) UMAP plots for the cell type identification in LNM and NLNM. (C) Heatmap showing marker genes for 6 distinct cell types. (D) Heatmap showing 6 distinct T-cell types proportional change in LNM and NLNM. (E) Analyzing the pseudotime trajectory and gene expression patterns of T-cell subtypes in LNM and NLNM. (F) Analyzing the pseudotime plot of cancer cell clusters. (G) Biomarkers of cancer cell clusters based on Pseudotime analysis. (H) Differences in pathway activity (scored per cell by GSVA) in 6 malignant cell subclusters. (I) Investigating the activation of transcription factors and molecular signatures in T-cell subtypes, as estimated using SCENIC. (J) Investigating the functional pathways (scored per cell by GSVA) in 5 malignant cell subclusters. LNM, lymph node metastasis; NLNM, non-lymph node metastasis-associated.

We performed UMAP clustering on T cells and identified six distinct subtypes of T cells, including proliferation T cells, CD8+ exhausted T cells (CD8 Tex), CD8+ tissue-resident memory T cells (CD8 Trm), naive T cells, and Tregs. Notably, T-cell content was significantly lower in samples with LNM, suggesting a potential T-cell desert phenomenon (Fig. 6A-B). We further classified T cells into five subclusters based on marker genes, including Naive T cells, CD8+ exhausted T cells, CD8+ tissue-resident memory T cells, proliferating T cells, and Tregs (Fig. 6C). In patients without LNM, proliferating T cells, which play an anti-tumor role in TME, were predominant. Conversely, in patients with LNM, CD8 Trm cells were predominant (Fig. 6D).

We conducted pseudotime analysis to order T cells in pseudotime, revealing their developmental trajectories. Clusters exhibited a relative time process, starting with proliferating T cells and ending with Treg and Naive T cells (Fig. 6E-F). This suggests a shift in the T-cell state from proliferation to non-functionality, with Treg cells and Naive T cells highly enriched in the final phase. Additionally, we found that all T-cell types were significantly reduced in LNM. Moreover, the expression of specific genes such as HIST1H4C, HMGB2, MKI67, NUSAP1, STMN1, TOP2A, UBE2C, and TUBA1B increased with T-cell evolution (Fig. 6G).

Furthermore, using single-cell regulatory network inference and clustering (SCENIC) analysis, we determined the underlying transcription factors of T cells. Transcription factors were found to be more active in patients with PTC and LNM, particularly in CD8 Trm, proliferating T cells, and Tregs (Fig. 6H). Gene set variation analysis (GSVA) revealed distinct molecular signatures for these T-cell subclusters, including cell-cycling signature, E2F targets, and G2M checkpoint for proliferating T cells; TNF-alpha signaling via NF-KB and cancer stem cell signature for CD8 Trm; hedgehog signature for CD8 Tex; and oxidative phosphorylation for Naive T cells (Fig. 6I-J). Each T-cell subtype displayed unique gene enrichment patterns related to specific cellular functions and signaling pathways, indicating their distinct roles in the immune response within the TME.

### Diminished B-cell types in papillary thyroid carcinoma with lymph node metastasis

**Figure 7:**
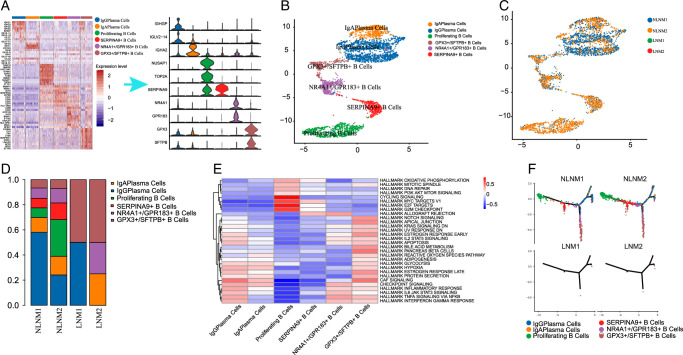
Diminished B-cell subtypes in papillary thyroid cancer tumor microenvironment with LNM. (A) UMAP plots for the cell type identification of 6 different B-cell types based on marker genes. (B) UMAP plots for the cell type identification in LNM and NLNM. (C) Heatmap showing marker genes for 6 distinct cell types. (D) Heatmap showing 5 distinct B-cell types proportional change in LNM and NLNM. (E) Investigating the functional pathways (scored per cell by GSVA) in 6 malignant cell subclusters. (F) Analyzing the pseudotime trajectory and gene expression patterns of T-cell subtypes in LNM and NLNM. LNM, lymph node metastasis; NLNM, non-lymph node metastasis-associated).

We identified three types of B cells within tumor-infiltrating cells based on marker expression: SERPINA9+, NR4A1+/GPR183+, and GPX3+/SFTPB+ B cells. Additionally, we annotated proliferating B cells, IgA effector B cells, and IgG effector B cells (Fig. 7A-B). Interestingly, B cells appeared to be diminished in patients with LNM, indicating a potentially crucial role in LNM (Fig. 7C). Half of the tumor-infiltrating B cells in LNM were GPX3+/SFTPB+ B cells (Fig. 7D).

By quantifying the tumor hallmark pathway using gene set enrichment analysis (GSEA) (Fig. 7E), we found that NR4A1+/GPR183+ and GPX3+/SFTPB+ B cells were more closely related to IgG effector B cells in terms of functional pathways, indicating their likely maturity. On the other hand, SERPINA9+ B cells showed similar functional pathways as proliferating B cells, suggesting that they could be immature. Moreover, we observed that proliferating B cells were more active in the cell cycle, E2F transcription factor pathway, and G2M checkpoint pathway, reflecting their proliferative properties. Although excessive B-cell activating factor can induce autoimmune diseases through proliferating B cells, emerging evidence suggests its crucial role in anti-tumor immunity. Based on these findings, we hypothesize that GPX3+/SFTPB+ B cells may inhibit LNM in PTC.

We conducted pseudotime analysis to project the developmental trajectories of B cells. Non-lymph node metastatic clusters exhibited a relative time process, starting with proliferating B cells and ending with IgG effector B cells (Fig. 7F). However, all B-cell types were significantly reduced in lymph node metastases, suggesting that PTC may be a “cold” tumor. We also identified several genes that promoted or suppressed proliferating B-cell development.

Additionally, we investigated the differentially expressed genes in these B-cell subtypes and performed GO enrichment analysis for up-regulated and down-regulated genes Supplementary Figure S3A-H, Supplemental Digital Content 2, http://links.lww.com/JS9/D57. Each B-cell subtype exhibited unique gene enrichment patterns related to specific cellular functions and signaling pathways. In summary, our study explored the types and roles of B cells in PTC with LNM. These findings shed light on the importance of B cells in tumor immunity and highlight the potential for targeting specific B-cell subtypes to improve patient outcomes.

### Predominant association of ATF3+/FOSB+/CXCL8+ CAFs in papillary thyroid carcinoma with lymph node metastasis

**Figure 8:**
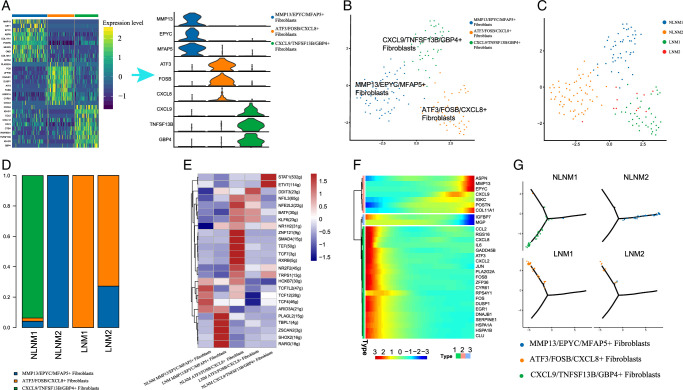
Predominant association of ATF3+/FOSB+/CXCL8+ CAFs in papillary thyroid cancer with LNM. (A) UMAP plots for the cell type identification of 3 cancer-associated fibroblasts based on marker genes. (B) UMAP plots for the cell type identification in LNM and NLNM. (C) Heatmap showing marker genes for 3 distinct cell types. (D) Heatmap showing 3 distinct fibroblast cells proportional change in LNM and NLNM. (E) Violin plots showing the expression of marker genes in 3 distinct fibroblast cells. (F) Heatmap of the t-value for the area under the curve score of expression regulation by transcription factors, as estimated using SCENIC. (G) Analyzing the pseudotime trajectory and gene expression patterns of CAF subtypes in LNM and NLNM. LNM, lymph node metastasis; NLNM, non-lymph node metastasis-associated).

We successfully classified CAFs into three types based on DEGs and marker genes displayed in the heatmap. These types included MMP13+/EPYC+/MFAP5+ CAFs, ATF3+/FOSB+/CXCL8+ CAFs, and CXCL9+/TNFSF13B+/GBP4+ CAFs. Notably, we found that ATF3+/FOSB+/CXCL8+ CAFs were predominantly associated with lymph node metastatic samples (Fig. 8A-D). We also observed key gene expression changes during the evolution of thyroid cells into tumor cells. Several genes showed increased expression during tumor development, including MMP13, POSTN, and EPYC, while the expression of DNAJB1, FOS, HSPA1A, PLA2G2A, and ZFP36 was negatively correlated (Fig. 8E).

To further explore the evolution of tumor cells, we conducted a pseudo-chronological analysis of tumor cell populations. In non-lymph node metastatic samples, ATF3+/FOSB+/CXCL8+ and CXCL9+/TNFSF13B+/GBP4+ CAFs developed into MMP13+/EPYC+/MFAP5+ CAFs (Fig. 8F-G). This analysis provides insights into the dynamic changes and evolution of CAFs during tumor progression in PTC.

### Distinct macrophage subtypes and their roles in papillary thyroid carcinoma with lymph node metastasis

**Figure 9:**
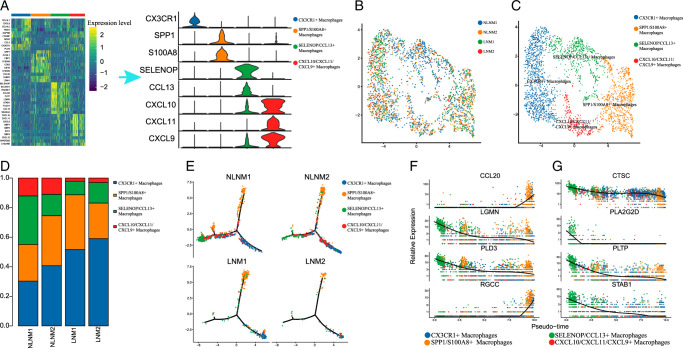
Transcriptomic heterogeneity of macrophages in papillary thyroid cancer tissues. (A) UMAP plots for the cell type identification of 4 macrophages. (B) UMAP plots for the macrophages identification in LNM and NLNM. (C) Heatmap showing marker genes for 4 distinct cell types. (D) Heatmap showing 4 distinct fibroblast cells proportional change in LNM and NLNM. (E) Violin plots showing the expression of marker genes in 4 distinct macrophages. (F) Analyzing the pseudotime trajectory and gene expression patterns of macrophages subtypes in LNM and NLNM. (G) Biomarkers of cancer cell clusters based on Pseudotime analysis. LNM, lymph node metastasis; NLNM, non-lymph node metastasis-associated).

Through differential analysis of four macrophage subtypes, we identified genes that were highly expressed only in specific macrophage subpopulations, which may serve as novel biomarkers for each type of macrophage (Fig. 9A). These macrophages were further categorized into four mechanical clusters: CX3CR1+ macrophages, SPP1+/S100A8+ macrophages, SELENOP+/CCL13+ macrophages, and CXCL10+/CXCL11+/CXCL9+ macrophages. The UMAP clustering and heatmap analysis displayed the differences in gene expression among these macrophage subpopulations (Fig. 9B-C).

Notably, CX3CR1+ macrophages were predominantly detected and accounted for the majority of macrophages in papillary thyroid carcinoma with lymph node metastasis (Fig. 9D). Furthermore, we performed GO enrichment analysis on the genes in module 3 and found evidence suggesting that SELENOP+/CCL13+ macrophages may inhibit the metastasis of BRAF-mutation-driven PTC cells through T-cell proliferation and receptor activation pathways Supplementary Figure S3A-H, Supplemental Digital Content 2, http://links.lww.com/JS9/D57. Different macrophage subtypes were also associated with specific gene modules. For example, CX3CR1+ macrophages were predominantly enriched in gene modules 1 and 4, SPP1+/S100A8+ macrophages in gene modules 6 and 10, SELENOP+/CCL13+ macrophages in gene module 3, 5, and 8, and CXCL10+/CXCL11+/CXCL9+ macrophages in gene modules 7 and 9 Supplementary Figure S5B, Supplemental Digital Content 2, http://links.lww.com/JS9/D57. Gene module 3 was particularly enriched in SELENOP+/CCL13+ macrophages http://links.lww.com/JS9/D57.

Pseudotime analysis revealed the evolutionary trajectories of macrophages. In PTCs with LNM, monocytes mainly differentiated into CX3CR1+ macrophages and SPP1+/S100A8+ macrophages. In non-lymph node metastatic PTCs, monocytes evolved towards SELENOP+/CCL13+ macrophages and CXCL10+/CXCL11+/CXCL9+ macrophages (Fig. 9E). The expression of CCL20 and RGCC increased with the evolution of macrophages, while the expression levels of LGMN, PLD3, CTSC, PLA2G2D, PLTP, and STAB1 were negatively correlated (Fig. 9F). These findings provide insights into the characterization and evolution of macrophage subpopulations in PTC, highlighting their distinct gene expression profiles and potential roles in tumor progression and metastasis.

### Development of multi-modal multi-tasks model for papillary thyroid carcinoma: advancing lymph node metastasis discrimination and prognostication

**Figure 10:**
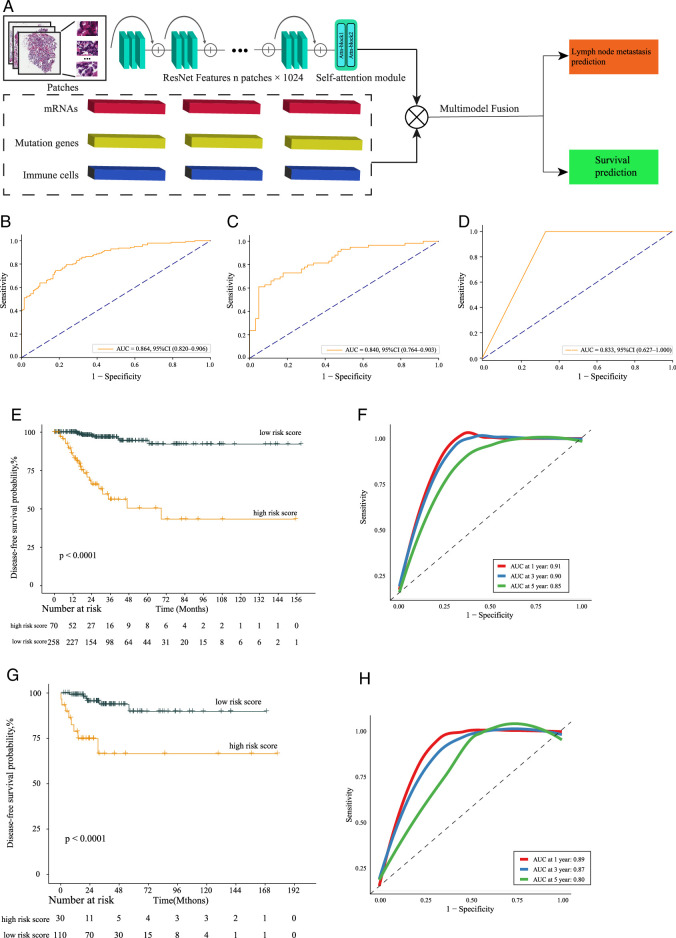
Algorithm construction and performance on cohorts. (A) Overview of the proposed algorithm. (B) ROC for predicting lymph node metastasis in The Cancer Genome Atlas (TCGA) training group. (C) ROC for lymph node metastasis in the TCGA validation group. (D) ROC for lymph node metastasis in the testing group of cohort 2. (E) Kaplan–Meier survival curve of the validation group. (F) ROC for the validation group. (G) Kaplan–Meier survival curve for the training group. (H) ROC for the training group for Assessing the model’s prognostic capabilities for disease-free survival. AUC, area under the curve; ROC, receiver operating characteristic curve).

We have developed a novel multi-modal multi-tasks deep-learning model that effectively integrates features from WSI, genomic data, transcriptomic data, immune cells, and is built upon the pre-trained ResNet50 model. This model possesses a self-attention module specifically for processing WSI, capable of automatically identifying and extracting the most effective areas for risk analysis, leading to more accurate identification of LNM and a better assessment of DFS in PTC patients. By utilizing this dual-pathway model, the system can integrate and cross-reference information from these two streams. This model integrates 586 WSI features and 1,004 mRNA expression profiles obtained from DEGs in a comparative analysis of two PTC patients with LNM and two PTC patients NLNM, which used scRNA-seq analysis, 22 mutated genes and the expression levels of 64 types of immune cells as found in the TCGA cohort (Fig. 10A).

A training cohort consisting of 405 pathology slides and RNA sequences from 328 PTC patients in the TCGA cohort was used for model training. For validation, a cohort comprising 181 slides and RNA sequences from 140 PTC patients was utilized. The model demonstrated high accuracy in predicting LNM, achieving an area under the curve (AUC) of 0.86 in the training cohort and 0.84 in the validation cohort (Fig. 10B-C), and an AUC of 0.83 in cohort 2 (Fig. 10D).

By integrating single-cell sequencing data with risk scores derived from pathology slides, mRNAs, mutation genes, and immune cells, the model effectively stratified PTC patients into high-risk and low-risk groups. In the training cohort, the high-risk group exhibited significantly lower DFS rates compared to the low-risk group (*P*<0.001) (Fig. 10E). The model’s accuracy, measured by AUC, was 0.91 at 1 year, 0.93 at 3 years, and 0.87 at 5 years (Fig. 10F).

The prognostic stratification capability of the model was further validated in an independent cohort. High-risk patients exhibited significantly lower DFS compared to low-risk patients (*P*<0.001) (Fig. 10G). The ROC curves indicated strong model performance, with AUCs of 0.89, 0.87, and 0.80 at 1, 3, and 5 years, respectively (Fig. 10H). These results underscore the robust performance of our multi-modal model and highlight its potential utility in PTC diagnosis, differentiation of LNM, and deeper understanding of disease mechanisms.

### Visualization and interpretation

In this study, we focused on the image regions and significant features influencing the output of the pathology-based deep-learning model. This effort aimed to enhance clinicians’ understanding of the network’s predictions and provide insights into tumor zones. We used the GradCAM algorithm to investigate the correlation between prediction capacity and deep-learning features in whole-slide images (WSIs). This technique transformed attention feature maps into visually informative heatmaps, offering an interpretable view of the model’s focal areas.

**Figure 11:**
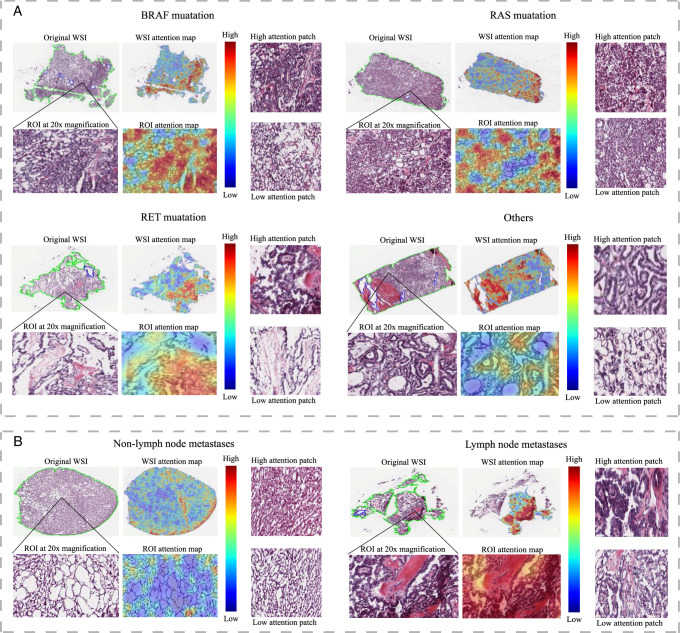
Histopathological analysis and attention mapping of tumor samples with various mutations and metastasis status. (A) BRAF mutation, RAS mutation, RET mutation and others. (B) Non-lymph node metastasis and lymph node metastasis.

The heatmaps generated in our analysis clearly highlighted areas of heightened response within the tumor tissues. Warm colors (e.g. red) indicated high influence, while cool colors (e.g. blue) represented a lower influence on the model’s predictions. Darker shades signified a stronger network response with higher attention weights, highlighting the model’s intensified focus on those specific areas. Attention maps in blue shades primarily depicted structural aspects of the tumors, such as boundaries, shapes, and textures. In contrast, red-biased attention maps predominantly captured high-level semantic tumor traits derived from anatomical images, along with functional insights. The results show that in pathological slides of PTC patients with different mutation types, various high-risk areas are visualized using image visualization techniques (Fig. 11A). Furthermore, when comparing pathological slides of patients with lymph node metastasis and those without, the results revealed that the pathological slides of PTC patients with lymph node metastasis exhibited a larger extent of high-risk areas (Fig. 11B).

This study’s visualizations offer clinicians critical insights into tumor zones, enhancing the interpretability of deep-learning model predictions in pathology. The ability to pinpoint high-risk areas, especially in cases with lymph node metastasis, could significantly improve diagnostic accuracy and treatment planning.

## Discussion

The present study aimed to comprehensively analyze the characteristics of PTC patients, including genetic alterations, molecular subgroups, immune microenvironment and survival outcomes. It categorizes PTC into four molecular subtypes based on mutations in key genes, each having a unique TME that adds to PTC’s complexity. It finds a significant link between BRAF mutations and LNM, affecting DFS and highlighting the importance of genetic alterations in PTC’s spread to lymph nodes and patient prognosis. The study employs scRNA-seq to investigate different T-cell subtypes and their roles in the TME, revealing a reduced diversity in B cells within BRAF-mutated PTC with LNM and examining the variety of fibroblasts and macrophages associated with LNM. For clinical decision-making, a novel deep-learning model integrating diverse data sources has been developed to predict preoperative LNM and assist in risk stratification in PTC. These insights gleaned from the study are instrumental in enhancing our understanding of PTC’s pathogenesis, with consequential impacts on its diagnosis, treatment approaches, and prognosis determination.

Gene mutations, particularly BRAF, play a pivotal role in the development, progression, and lymph node metastasis potential of PTC, contributing to a more aggressive disease course^18^. Our study aligns with previous research, emphasizing the significant role of BRAF mutation in enhancing PTC aggressiveness and LNM-related mortality^18^. We observed that BRAF mutations, overactivating the MAPK pathway, are associated with invasive features in smaller PTCs (≤1.5 cm) and high-risk histological subtypes. Additionally, our investigation into mutation heterogeneity, including BRAF, RAS, and RET mutations, revealed a strong association of BRAF mutations with LNM and unfavorable prognoses in cases with LNM^19–21^. The study also explored immune cell infiltration disparities among PTC subtypes, providing valuable insights into mutation heterogeneity and its implications in disease progression and treatment. In PTC with lymph node metastasis, especially those with BRAF mutations, we observed the immune desert phenomenon, marked by diminished T-cell infiltration, impacting patient prognosis^22,23^. Our research underscores the need for refined prognostic models and emphasizes the essential role of immune microenvironment variations in tailoring immunotherapeutic strategies for PTC.

Two well-known classes are classically activated M1 macrophages and alternatively activated M2 macrophages, distinguished by activation states, functions, and secreted factors^24^. Recent research identified additional macrophage types, such as FAP+ fibroblasts and SPP1+ macrophages in colorectal cancer tissue, forming a “wall-like” tumor border hindering T-cell infiltration^25^. In our study, we identified four macrophage types: CX3CR1+, SPP1+-S100A8+, SELENOP+-CCL13+, and CXCL11+. CX3CR1+ macrophages, associated with LNM in gastric cancer, were more prevalent in the LNM group, indicating a potential contribution to LNM in BRAF-mutated PTC patients. Additionally, CHI3L1+/S100A8+ macrophages, with negative impacts on anti-tumor immunity, were higher in the LNM group, albeit at a slightly lower proportion than CX3CR1+ macrophages. SELENOP+/CCL13+ and CXCL11+ macrophages exhibited high levels of chemokines CXCL9 and CXCL10, providing further insights into the intricate immune mechanisms in PTC progression.

Cancer-associated fibroblast classification plays an indispensable role in regulating the occurrence and development of PTC. CAFs shape TME by secreting cytokines, growth factors, chemokines, and exosomes, creating an immunosuppressive milieu that allows cancer cells to evade immune surveillance^26^. CAF isoforms exhibit complex dual functions, with both pro-tumor and anti-tumor activities evolving during cancer progression^24,25^. Clearance of CAFs in a pancreatic ductal adenocarcinoma mouse model resulted in poorly differentiated tumors and reduced survival times, indicating that sonic-hedgehog-driven CAFs can inhibit tumor growth and progression^27,28^. Recently, CD146+/CAV1-CAF and PDGFRα+ CAF were identified as tumor suppressor CAF subsets in breast cancer^29^. In this study, we performed CAF classification on patients with lymph node metastatic PTC, and non-lymph node metastatic PTC was mainly infiltrated by MMP13+/EPYC+/MFAP5+ CAF and CXCL9+/CCL5+/ TNFSF13B+/ GBP4+ CAF. Lymph node metastatic PTC was mainly infiltrated by MMP13+/ EPYC+/ MFAP5+ CAF.

Artificial intelligence breakthroughs, especially in the realm of medical image processing, have ushered in a paradigm shift in cancer detection and evaluation, spanning diverse cancer types such as breast, lung, and colon cancers^30–33^. In the context of PTC, where precise LNM detection is pivotal for informed surgical decisions and accurate prognosis, we leveraged AI to pioneer a multi-modal, dual-track prognostic model. By incorporating pathology slide features, mRNA expression, mutation genes, and immune cell data, the model signifies a substantial advancement in predicting LNM and delivering precise prognostication. Its commendable accuracy and ability to differentiate between high- and low-risk patient groups underscore its clinical significance, elevating precision and dependability in risk assessments. The model’s distinctive strength lies in its simultaneous analysis of multiple tasks, embracing a multi-tasking framework that enhances its versatility. The amalgamation of diverse data types empowers the model to formulate tailored treatment plans, thereby improving patient outcomes and highlighting the indispensable role of AI in personalized cancer care. This groundbreaking model excels in predicting outcomes for thyroid cancer, positioning itself as a pivotal tool in guiding the management of PTC patients.

AI-clustering techniques marks a significant step forward in computational pathology, offering new avenues for research and clinical practice in the management of cancer^34^. This study delves into the diverse morphological and molecular behaviors of PTC through advanced image analysis and deep-learning models. We employed the GradCAM algorithm to generate heatmaps that visually interpret the deep-learning model’s focus areas within WSIs. By highlighting areas with different degrees of influence, it helps to elucidate the model’s predictions. By incorporating these visual and interpretative tools, we aim to bridge the gap between molecular data and morphological features, enhancing the clinical utility of our findings. By linking molecular findings to specific morphological subsets, our research aims to provide a more precise and clinically useful framework. The integration of AI-driven clustering and visualization techniques offers a promising approach to diagnose and treat PTC more accurately. These advancements not only improve interpretability but also potentially lead to more personalized and effective treatment plans, ultimately improving patient outcomes.

## Limitation

One of the primary limitations of our study is while our research provides a comprehensive analysis of molecular subtypes based on genetic alterations, it does not integrate pathological morphological subtyping, which could broaden the model’s applicability. Future studies should incorporate both molecular and morphological data to enhance the robustness and generalizability of our predictive model, ensuring it can be effectively utilized in diverse clinical settings. Additionally, the model’s performance should be assessed across various patient cohorts to confirm its accuracy and reliability in predicting LNM, DFS and informing clinical decision-making in prospective studies.

## Conclusions

In conclusion, this study unveils the molecular and TME heterogeneity of PTC and introduces an AI-based multi-modal analysis to predict preoperative LNM and prognosis. AI-based analysis categorizes PTC, revealing four molecular subtypes and unique TMEs. BRAF mutations correlate significantly with LNM, impacting DFS. A deep-learning model integrating diverse data accurately predicts preoperative metastasis and risk in PTC with high accuracy. This comprehensive approach offers valuable insights into PTC heterogeneity, immune microenvironment, and potential avenues for improved diagnostic and therapeutic strategies.

## Ethical approval

The study protocol was approved by the ethics committee of each participating hospital and was conducted in accordance with the Declaration of Helsinki and Good Clinical Practice guidelines. The relevant Judgement's reference number is SYSEC-KY-KS-2021-259, the name of the committee is “The Ethic Commitee of Sun Yat-Sen Memorial Hospital of Sun Yat-Sen University”

## Consent

The requirement for informed consent from patients whose information was retrospectively collected from participant hospitals was waived.

## Source of funding

This study was supported by grants 2023YFE0204000 from National Key R&D Program of China, grants 2023B1212060013 from the Science and Technology Planning Project of Guangdong Province, grants 82273204 from the National Natural Science Foundation of China, grant 2023A1515012412 and 2023A1515011214 GuangDong Basic and Applied Basic Research Foundation, grant 2023A03J0722, 202206010078, 202201020574, and 2024A03J1194 from the Guangzhou Science and Technology Project, grant 7670020025 from Tencent Charity Foundation, grant YXQH202209 from the Sun Yat-sen Pilot Scientific Research Fund.

## Author contribution

Y.Y., W.O. and Y.H. are co-first authors. H.Y. and M.L. are co-senior authors. Concept and design: H.Y. and M.L. Acquisition, analysis, or interpretation of data: all authors. Drafting of the manuscript: all authors. Critical revision of the manuscript for important intellectual content: all authors. Statistical analysis: X.L., F.Z., Z.C. Obtained funding: M.L., H.Y. Administrative, technical, or material support: Y.Y., H.Y. Supervision: M.L., H.Y.

## Conflicts of interest disclosure

The author declares no conflicts of interest.

## Research registration unique identifying number (UIN)

1. Registry used: Y. Yu.
2. Unique Identifying number or registration ID：NCT06241092.
3. Hyperlink to your specific registration (must be publicly accessible and will be checked) https://www.clinicaltrials.gov/
4. Org: SunYatsenU2H.

## Guarantor

Miaoyun Long.

## Data availability statement

For data resources, please contact the corresponding author. All data reported in this paper will be shared by the lead contact upon request. This paper does not report original code. Any additional information required to reanalyze the data reported in this paper is available from the lead contact upon request.

## Provenance and peer review

Not commissioned, externally peer-reviewed.

## Materials availability

This study did not generate new unique reagents.

## Supplementary Material
