# Supplementary material for: Artificial intelligence-based multi-modal multi-tasks analysis reveals tumor molecular heterogeneity, predicts preoperative lymph node metastasis and prognosis in papillary thyroid carcinoma: a retrospective study
Source: Int J Surg. 2024 Jul 11;111(1):839–56. doi: 10.1097/JS9.0000000000001875 (PMC11745641; doi:10.1097/JS9.0000000000001875)
Supplement: Supplementary file 2 [file js9-111-0839-s002.docx]

**Supplementary Content**

**Supplementary Methods**

**Supplementary Table1. Summary of enrolled patients of mutation sequencing in this study**

**Supplementary Table 2. Top 18 common mutated gene of PTCs**

**Supplymentary figure 1. relationship between molecular subtypes and clinicopathological features. Comparation of age in SYSMH and TCGA cohort.**

**Supplymentary figure 2. Comparison of disease-free survival between patients with LNM and NLNM.**

**Supplymentary figure 3. Enrichment analysis of B cell of related marker gene.**

**Supplymentary figure 4. Analysis of differences expressed genes in Macrophage between lymph node metastases and non-lymph node metastases.**

**Supplymentary figure 5. Biomarkers of macrophage clusters and hotspot module analysis.**

**Supplementary Methods**

**Sequencing Data Acquisition and Processing**

**DNA isolation and capture-based targeted DNA sequencing**

DNA isolation and targeted sequencing were performed at Burning Rock Biotech, a commercial clinical laboratory accredited by the College of American Pathologist (CAP) and certified by the Clinical Laboratory Improvement Amendments (CLIA), according to the optimized protocols described previously (16, 17). Briefly, tissue DNA was extracted from FFPE tumor tissues using the QIAamp DNA FFPE tissue kit according to the manufacturer’s instructions (Qiagen, Hilden, Germany). The DNA concentration was measured by Qubit dsDNA assay (Life Technologies, California, US). For NGS library preparation, DNA was subjected to end repair, phosphorylation, and adaptor ligation. Fragments measuring 200–400 bp in size were selected by beads (Agencourt AMPure XP Kit, Beckman Coulter, CA, USA), followed by hybridization with capture probe panels consisting of 18 PTC-related genes (Supplementary Table 1), hybrid selection with magnetic beads, and PCR amplification. The panel comprised 18 genes that are closely relevant to the pathogenesis and development of PTC. The Agilent 2100 Bioanalyzer (Agilent Technologies) was used to assess the QC of the fragments. Indexed samples were deep-sequenced on the Nextseq500 sequencer (Illumina, Inc., US) with pair-end reads. The sequence data were mapped to the human genome (hg19) using BWA Aligner 0.7.10 (18).

**Dissociated single cell suspension of tissue**

The fresh tissues were stored in the sCelLiveTM Tissue Preservation Solution (Singleron Bio Com, Nanjing, China) on ice after the surgery within 30 mins. Washed the PTC tissues three times with Hanks Balanced Salt Solution (HBSS), cut into 1-2mm pieces, digest and dissociate with 2ml of GEXSCOPETM dissociation solution (Singleron) at 37°C for 15 minutes, during which shaking is required. After digestion, filter the samples through a 40-micron sterile filter and centrifuge the filtrate for five minutes at 1000 rpm. Discard the supernatant and resuspension all cells in 1 ml of PBS (HyClone). To remove too many erythrocytes, add 2 mL of GEXSCOPETM erythrocyte lysis buffer (Singleron) and let it stand for 10 minutes at 25°C. Centrifuge at 500g for 5 minutes, resuspend the cells in PBS, then stain with trypan blue (Sigma), and count the number of viable cells under the microscope.

**Single Cell Transcriptome Library Sequencing**

The four PTC tissues were made a single cell suspension in PBS (HyClone) at a concentration of 1×10^5^ cells/mL. Apply the single-cell suspension to the microfluidic chip and use the GEXSCOPETM Single-Cell RNA Library Kit (Singleron Biotechnologies) to construct a scRNA-seq library according to the Singleron GEXSCOPETM operating instructions. The library was diluted to 4nM, then sequenced using an Illumina novaseq 6000 sequencing platform in a paired-end mode with 150 bp reads.

**Primary analysis of raw read data**

Raw reads from scRNA-seq were processed to generate gene expression matrixes using CeleScope (https://github.com/singleron-RD/CeleScope) v1.4.0 pipeline. Briefly, raw reads were first processed with CeleScope to remove low quality reads with Cutadapt v1.17 (19)to trim poly-A tail and adapter sequences. Cell barcode and UMI were extracted. After that, we used STAR v2.6.1a (20) to map reads to the reference genome GRCh38 (ensembl version 92 annotation). UMI counts and gene counts of each cell were acquired with featureCounts v2.0.1 (21) software, and used to generate expression matrix files for subsequent analysis.

**Quality control, dimension-reduction and clustering**

Cells were filtered by gene counts below 200 and the top 2% gene counts and the top 2% UMI counts. Cells with over 20% mitochondrial content were removed. After filtering, 27,351 cells were retained for the downstream analyses, with on average 2108.319 genes and 8085.971 UMIs per cell. We used functions from Seurat v3.1.2 (22) for dimension-reduction and clustering. Then we used NormalizeData and ScaleData functions to normalize and scale all gene expression, and selected the top 2000 variable genes with FindVariableFeautres function for PCA analysis. Using the top 20 principle components, we separated cells into multiple clusters with FindClusters. Batch effect between samples was removed by Harmony (23). Finally, UMAP algorithm was applied to visualize cells in a two-dimensional space.

**Single Cell Sequencing Data Generation** **and Analysis**

**Differentially expressed genes analysis**

To identify differentially expressed genes (DEGs), we used the Seurat FindMarkers function based on Wilcox likelihood-ratio test with default parameters, and selected the genes expressed in more than 10% of the cells in a cluster and with an average log (Fold Change) value greater than 0.25 as DEGs. For the cell type annotation of each cluster, we combined the expression of canonical markers found in the DEGs with knowledge from literatures, and displayed the expression of markers of each cell type with dot plots that were generated with Seurat DotPlot function. Doublet cells were identified as expressing markers for different cell types, and removed manually.

**Cell type annotation**

The cell type identity of each cluster was determined with the expression of canonical markers found in the DEGs using SynEcoSys database. Heatmaps/dot plots/violin plots displaying the expression of markers used to identify each cell type were generated by Seurat v3.1.2 DotPlot.

**Pathway enrichment analysis**

To investigate the potential functions of DEGs, the Gene Ontology (GO) and Kyoto Encyclopedia of Genes and Genomes (KEGG) analysis were used with the “clusterProfiler” R package 4.0.2 (24). Pathways with p_adj value less than 0.05 were considered as significantly enriched. Gene Ontology gene sets including molecular function (MF), biological process (BP), and cellular component (CC) categories were used as reference.For GSVA pathway enrichment analysis, the average gene expression of each cell type was used as input data using the GSVA package 1.44.2 (25).

**scRNA-seq based CNA detection**

The InferCNV package was used to detect the CNAs in thyroid malignant cells (26). T cells, B cells, CAF, Macrophage and non-malignant cells were used as baselines to estimate the CNAs of malignant cells. Genes expressed in more than 20 cells were sorted based on their loci on each chromosome. The relative expression values were centered to 1, using 1.5 standard deviation from the residual-normalized expression values as the ceiling. A slide window size of 101 genes was used to smoothen the relative expression on each chromosome, to remove the effect of gene-specific expression

**Intra-tumoral heterogeneity score calculation**

The intra-tumoral heterogeneity score was calculated by the algorithm described in published work (27). The intra-tumoral heterogeneity score was defined as the average Euclidean distance between the individual cells and all other cells, in terms of the first 20 principal components derived from the normalized expression levels of highly variable genes. The highly variable gene was identified using the FindVariableFeatures function in the Seurat package, with default parameters.

**Trajectory analysis**

Cell differentiation trajectory was reconstructed with Monocle2 v2.22.0 (28). Highly-variable genes were used to sort cells in order of spatial‐temporal differentiation. We used DDRTree to perform FindVairableFeatures and dimension-reduction.

**Transcription factor regulatory network analysis**

Transcription factor network was constructed by pyscenic v0.11.0 (29) using scRNA expression matrix and transcription factors in AnimalTFDB. First, GRNBoost2 predicted a regulatory network based on the co-expression of regulators and targets. CisTarget was then applied to exclude indirect targets and to search transcription factor binding motifs. After that, AUCell was used for regulon activity quantification for every cell. Top TF regulons with high RSS (Regulon Specificity Score) were visualized using pheatmap in R.

**Functional Gene Module Analysis**

Hotspot was used to identify functional gene modules which illustrate heterogeneity within macrophage subpopulations. Briefly, we used the ‘danb’ model and selected the top 500 genes with highest autocorrelation zscore for module identification. Modules were then identified using the create_modules function, with min_gene_threshold =15 and fdr threshold=0.05. Module scores were calculated by using calculate_module_scores function.

**Jaccard similarity analysis**

The Jaccard similarity coefficient was calculated for comparing transcriptional similarity between two cell types using their signature genes. We evaluated transcriptional similarity between ten (30) meta-programs of macrophage cells and signatures of four cell types by calculated Jaccard similarity coefficients using the top 50 marker genes.

**Cell-cell interaction analysis (CellPhoneDB)**

The cell-cell interaction analysis was performed by CellPhoneDB v2.1.7 (31) based on known receptor–ligand interactions between two cell types/subtypes. Cluster labels of all cells were randomly permuted for 1000 times to calculate the null distribution of average ligand-receptor expression levels of the interacting clusters. Individual ligand or receptor expression was thresholded with a cutoff value based on the average log gene expression distribution for all genes across all the cell types. The significant cell-cell interactions were defined as p value < 0.05 and average log expression > 0.1, which were visualized with the circlize v0.4.10 R package.

**Deep learning model architecture and model evaluation**

Deep learning-based survival analysis and subtype prediction for integrating whole slide images and genomic features

MRAP (Multi-modal Residual Attention Prediction) employs a high-throughput, easily understandable, weakly-supervised, and multimodal deep learning algorithm (MMF) that is specifically devised for amalgamating whole slide images and molecular profile data in weakly-supervised learning tasks. These tasks include analyzing the prognosis of a patient's cancer at the patient-level via survival and subtype analysis. The MMF algorithm can learn to jointly represent two heterogeneous data modalities, namely diagnostic WSIs as pyramidal files, and processed genomic and transcriptomic features for a single patient. Our algorithm is not limited to survival and subtype analysis and can be adapted to any combination of those modalities. Additionally, our algorithm is flexible enough to handle multi-level learning tasks in computational pathology that have patient-level labels. The algorithm comprises three main components: 1) CLAM for processing WSIs, 2) Random Forest and Survival difference analysis for processing molecular profile data, and 3) a multimodal fusion layer for integrating WSIs and molecular profile data.

WSI processing

We employed advanced deep learning methodologies to accurately predict both the risk score and the probability of different subtypes simultaneously, and assess the survival analysis of every patient by integrating both WSIs and molecular features. Nevertheless, the use of deep learning for de novo processing of complete WSIs during training and processing can be suboptimal due to the increased pixel size of histopathological assets. To overcome this limitation, we initiated the process by utilizing the WSI processing techniques from CLAM [1], which enabled tissue segmentation and patching of each slide. This approach empowered us to enhance efficiency by retaining only the valid areas that would be cropped into multiple 256x256 RGB images without any overlap at the 20x equivalent pyramid level from all the identified tissue regions. Subsequently, we performed targeted feature extraction on each partitioned section using a pre-trained ResNet50 [2] model on ImageNet [3]. This process enabled us to capture the 1024-dimensional latent feature map from the low-dimensional space of each sub-region to the latent feature space via spatial average pooling after the 3rd residual block.

Attention gate

To perform survival prediction and subtype classification from WSIs, we have expanded the attention-based multiple instance learning algorithm that was initially proposed for weakly-supervised classification. As it can be demanding and time-consuming to provide intricate region annotations for pathological images, especially for WSIs that lack annotations, it becomes challenging to differentiate the importance of different regions for the final task. To overcome this obstacle, we adopted a state-of-the-art attention gate model [4], which can automatically extract the most efficient regions for risk analysis from the image. In the multiple instance learning framework, each gigapixel WSI is partitioned into smaller regions and considered as a collection (bag) of patches (instances) with a corresponding slide-level label used for training. To consolidate the information from different patches, we further filtered them through attention gates. To elaborate, since survival outcome information and subtype information are obtained at the patient level rather than on a single slide, during training and evaluation, we will process all $N$ WSIs corresponding to the patient cases as a single WSI bag collectively. Hence, the formula $\boldsymbol{A}=[\boldsymbol{M}_{1}|\boldsymbol{M}_{2}|...|\boldsymbol{M}_{N}]$can be utilized to denote all the pathological characteristics of the tissue samples $\boldsymbol{M} = \{\mathbf{M}_{i} | \mathbf{M}_{i}\in\mathbb{R}^{m_{i}\times1024} , i \in\{1,2,...,N\}\}$ of an individual, wherein the dimension of $\boldsymbol{M}$ is equivalent to the patch number $m_{i}$multiplied by 1024, while $i$ represents one of the slides belonging to the patient.

Specifically, the architecture of our model encompasses four cardinal constituents, namely the linear projection layer $f_{lp}$, the attention gate mechanism $f_{a}$, and the adaptable forecasting layer $f_{pre}$, which endows the product to be customized for classification or regression activities, targeted at survival analysis or subtype classification. In order to guarantee that all path-level feature $\boldsymbol{X} \in\mathbb{R}^{N\times1024}$embeddings of a patient can be modified according to the attention gate, the fully-connected layer $f_{lp}$ with weights $\boldsymbol{W}_{lp}$ and bias $\boldsymbol{b}_{lp}$ is utilized. Through this approach, the original feature map is projected into a 768-dimensional feature spacethat that can dynamically adjust and adapt to the attention gate, thereby augmenting the precision and efficiency of the model. Subsequently, the attention gate $f_{a}$ acquires the ability to score each region based on its perceived relevance to patient-level prognostic prediction. Regions with high attention scores are more contributive to the patient-level feature representation compared to regions allotted low attention scores, when information across all regions in the patient’s WSIs is accumulated, in an operation recognized as attention-pooling[1]. In $f_{a}$, there are three fully-connected layers with weights $\boldsymbol{Q}_{a} \in\mathbb{R}^{384\times768}, \boldsymbol{K}_{a} \in\mathbb{R}^{384\times768}, \boldsymbol{W}_{a} \in\mathbb{R}^{1\times384}$ (the bias terms are implied for succinctness), then the attention score $\boldsymbol{a}$ can be represent:

$$\boldsymbol{a}=\frac{exp\left\{ \boldsymbol{W}_{a}\left( tanh\left( \boldsymbol{Q}_{a}\boldsymbol{X} \right) \odot sigm\left( \boldsymbol{K}_{a}\boldsymbol{X} \right) \right) \right\}}{\sum_{i=1}^{N} exp\left\{ \boldsymbol{W}_{a}\left( tanh\left( \boldsymbol{Q}_{a}\boldsymbol{X}_{i} \right) \odot sigm\left( \boldsymbol{K}_{a}\boldsymbol{X}_{i} \right) \right) \right\}}$$

Subsequently, the attention-pooling operation consolidates the patch-level feature representations into the patient representation, $\boldsymbol{x}_{p}\in\mathbb{R}^{768}$, by leveraging computed attention scores as weight coefficients, where $\boldsymbol{A}\in\mathbb{R}^{A}$ is the vector of attention scores:

$$\boldsymbol{x}_{p}=Attn(\boldsymbol{A}, \boldsymbol{X}) = \sum_{i = 1}^{A} {\boldsymbol{a}_{i}\boldsymbol{X}}_{i}$$

The ultimate patient-level prognostication scores $h_{out}\in\mathbb{R}^{k\times1}$ are computed from the bag representation via the employment of the prediction layer $f_{pre}$ furnished with weights $\boldsymbol{W}_{pre} \in\mathbb{R}^{k\times768}$, where $k$ is ascertained by the task at hand. For subtype classification, $k$ is set to 4, representing the probability of all feasible subtypes, whereas for survival analysis, $k$ assumes a value of 1, serving to appraise the risk score. Thus, the concluding output shall be denoted as $h_{out}=f_{pre}$($\boldsymbol{x}_{p}$). Moreover, the last linear layer is designed to blend the patient representation $\boldsymbol{x}_{p}$ and the genomic features to facilitate survival analysis.

Integrating WSIs and genomic features

In view of the strong correlation between gene and immune cell expression and patient survival, we synchronously embedded gene and immune cell features after passing through the attention gate to enhance prediction accuracy. In the final fully-connected layer, the input of the entire genomic representation $\boldsymbol{x}_{g}$ will be merged with the patient representation $\boldsymbol{x}_{p}$:

$$\boldsymbol{x}_{fusion}=\boldsymbol{x}_{p}\bigoplus\boldsymbol{x}_{p}$$

Ultimately, we ascertained the risk score by utilizing a regressor, which incorporates a linear projection to attain an accurate assessment of disease-free survival.

**Supplementary Table1. Summary of enrolled patients of mutation sequencing in this study**

|  | **SYSMH (N=252)** | **TCGA (N=499)** | **P-value** | **total (N=751)** |
| --- | --- | --- | --- | --- |
| **Age** |  |  |  |  |
| Mean; SD | 42.1; 11.1 | 47.3; 15.8 | <0.001 | 45.6; 14.6 |
| Median [Min, Max] | 41.5 [10.0, 77.0] | 46.0 [15.0, 89.0] |  | 44.0 [10.0, 89.0] |
| **Gender** |  |  |  |  |
| Female | 188 (75 %) | 365 (73 %) | 0.734 | 553 (74 %) |
| Male | 64 (25 %) | 134 (27 %) |  | 198 (26 %) |
| **Stage** |  |  |  |  |
| I | 198 (79 %) | 282 (57 %) | <0.001 | 480 (64 %) |
| II | 20 (8 %) | 51 (10 %) |  | 71 (9 %) |
| III | 1 (0 %) | 110 (22 %) |  | 111 (15 %) |
| IV | 0 (0 %) | 54 (11 %) |  | 54 (7 %) |
| Missing | 33 (13.1%) | 2 (0.4%) |  | 35 (4.7%) |
| **T stage** |  |  |  |  |
| 1 | 101 (40 %) | 143 (29 %) | <0.001 | 244 (32 %) |
| 2 | 8 (3 %) | 166 (33 %) |  | 174 (23 %) |
| 3 | 108 (43 %) | 166 (33 %) |  | 274 (36 %) |
| 4 | 4 (2 %) | 22 (4 %) |  | 26 (3 %) |
| Missing | 31 (12.3%) | 2 (0.4%) |  | 33 (4.4%) |
| **N stage** |  |  |  |  |
| 0 | 123 (49 %) | 227 (45 %) | 0.715 | 350 (47 %) |
| 1 | 129 (51 %) | 222 (44 %) |  | 351 (47 %) |
| Missing | 0 (0%) | 50 (10.0%) |  | 50 (6.7%) |
| **M stage** |  |  |  |  |
| 0 | 220 (87 %) | 277 (56 %) | 0.153 | 497 (66 %) |
| 1 | 2 (1 %) | 9 (2 %) |  | 11 (1 %) |
|  | 0 (0 %) | 1 (0 %) |  | 1 (0 %) |
| Missing | 30 (11.9%) | 212 (42.5%) |  | 242 (32.2%) |
| **BRAF** |  |  |  |  |
| (-) | 40 (16 %) | 202 (40 %) | <0.001 | 242 (32 %) |
| (+) | 212 (84 %) | 297 (60 %) |  | 509 (68 %) |

| **Gene symbol** | **Descriptions** |
| --- | --- |
| AKT1 | AKT Serine/Threonine Kinase 1 |
| ALK | ALK Receptor Tyrosine Kinase |
| BRAF | B-Raf Proto-Oncogene |
| [CTNNB1](https://www.genecards.org/cgi-bin/carddisp.pl?gene=CTNNB1&keywords=CTNNB1) | Catenin Beta 1 |
| EIF1AX | Eukaryotic Translation Initiation Factor 1A X-Linked |
| ETV6 | ETS Variant Transcription Factor 6 |
| GNAS | GNAS Complex Locus |
| HRAS | HRas Proto-Oncogene |
| KRAS | KRAS Proto-Oncogene |
| NRAS | NRAS Proto-Oncogene |
| NTRK1 | Neurotrophic Receptor Tyrosine Kinase 1 |
| PIK3CA | Phosphatidylinositol-4,5-Bisphosphate 3-Kinase Catalytic Subunit Alpha |
| PPARG | Peroxisome Proliferator Activated Receptor Gamma |
| PTEN | Phosphatase And Tensin Homolog |
| RET | Ret Proto-Oncogene |
| TERT | Telomerase Reverse Transcriptase |
| TP53 | Tumor Protein P53 |
| TSHR | Thyroid Stimulating Hormone Receptor |

**Supplementary Table 2**

**Supplymentary figure 1. Relationship between molecular subtypes and clinicopathological features. Comparation of age in SYSMH and TCGA cohorts.**


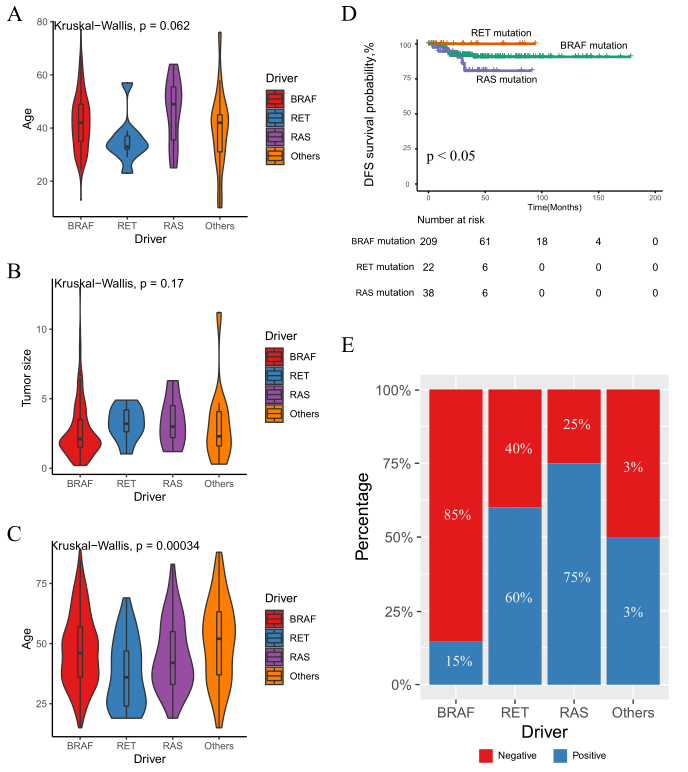


DFS Probability,%

(A) Age composition of four mutation types in BRAF-, RET-, RAS and other-driven tumors in SYSMH. The RET mutation population was significantly younger (Kruskal-Wallis, P<0.05) (B) Tumor size of four mutation types in BRAF-, RET-, RAS and other-driven tumors in SYSMH. Patients with RET and RAS mutations had larger average maximum diameters (Kruskal-Wallis, P<0.05). (C) Age composition of four mutation types in BRAF-, RET-, RAS and other-driven tumors in TCGA. The RET mutation population was significantly younger (Kruskal-Wallis, P<0.05). Clinicopathological characteristics and genes of SYSMH TCGA cohorts. (D) Comparison of disease-free survival between patients with nodal-negative in 4 molecular subtypes. RAS had a lower survival rate and RET had a higher survival rate. (P<0.05). (E) Precentage of TPO positive in 4 molecular subtypes (P<0.05) (SYSMH: Sun Yat-sen University; TCGA: The Cancer Genome Atlas)

**Supplymentary figure 2. Comparison of disease-free survival between patients with LNM and NLNM.**

**
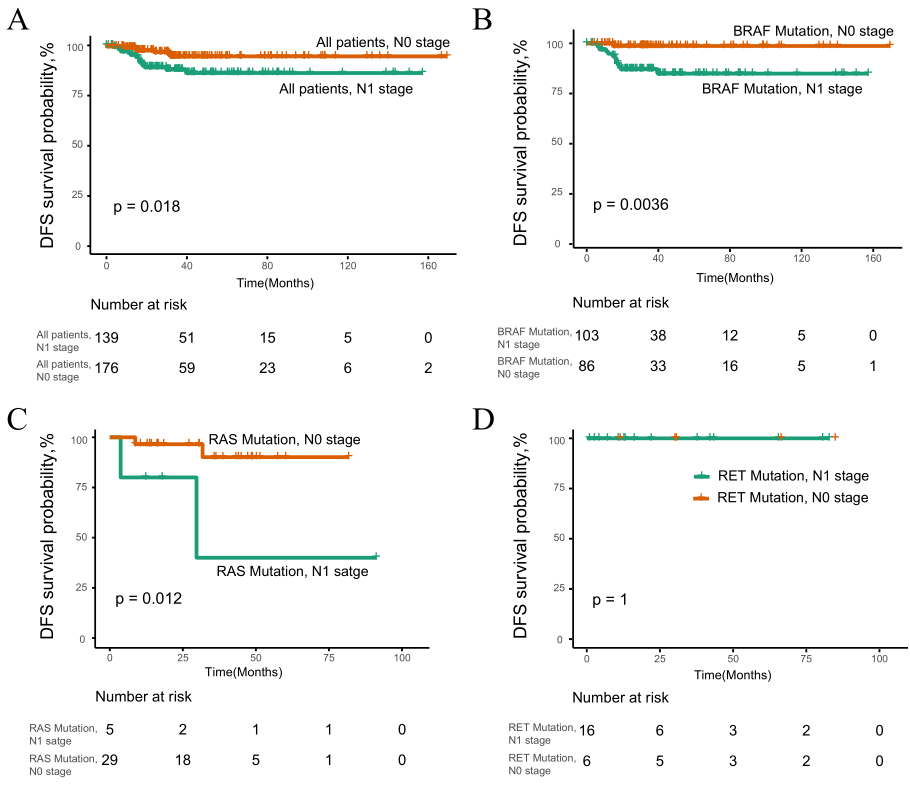
**

(A) All patients. (B)BRAF-mutant subgroup (C) RAS-mutant subgroup and (D)RET-mutant subgroup. (LNM: lymph node metastases; NLNM: non-lymph node metastases)

**Supplymentary figure 3. Enrichment analysis of B cell of related marker gene.**

**
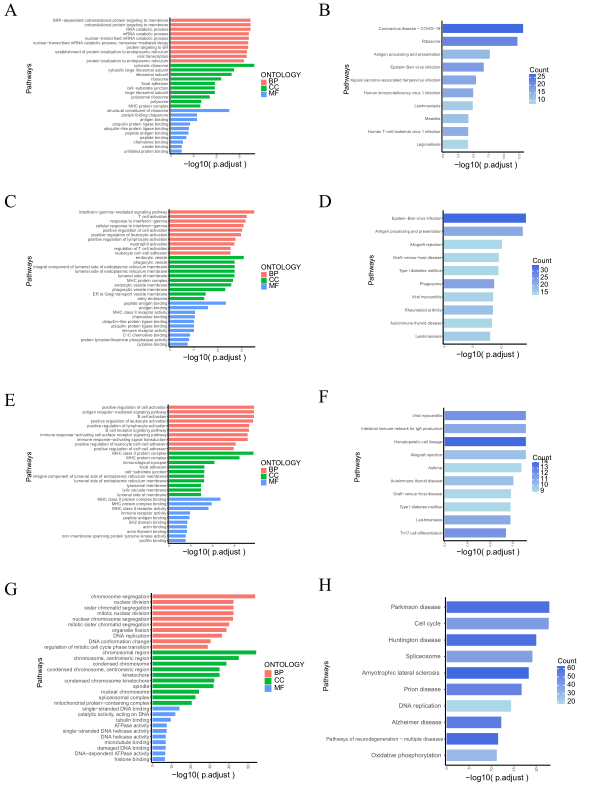
**

(A)Barplots showing the upregulated GO pathways of type 1 B cells. (B)Barplots showing the upregulated KEGG pathways of type 1 B cells. (C)Barplots showing the upregulated GO pathways of type 2 B cells. (D)Barplots showing the upregulated KEGG pathways of type 2 B cells. (E)Barplots showing the upregulated GO pathways of type 3 B cells. (F)Barplots showing the upregulated KEGG pathways of type 3 B cells. (G)Barplots showing the upregulated GO pathways of type Proliferating B cells. (H)Barplots showing the upregulated KEGG pathways of Proliferating B cells.

**Supplymentary figure 4. Analysis of differences expressed genes in Macrophage between LNM and NLNM.**

**
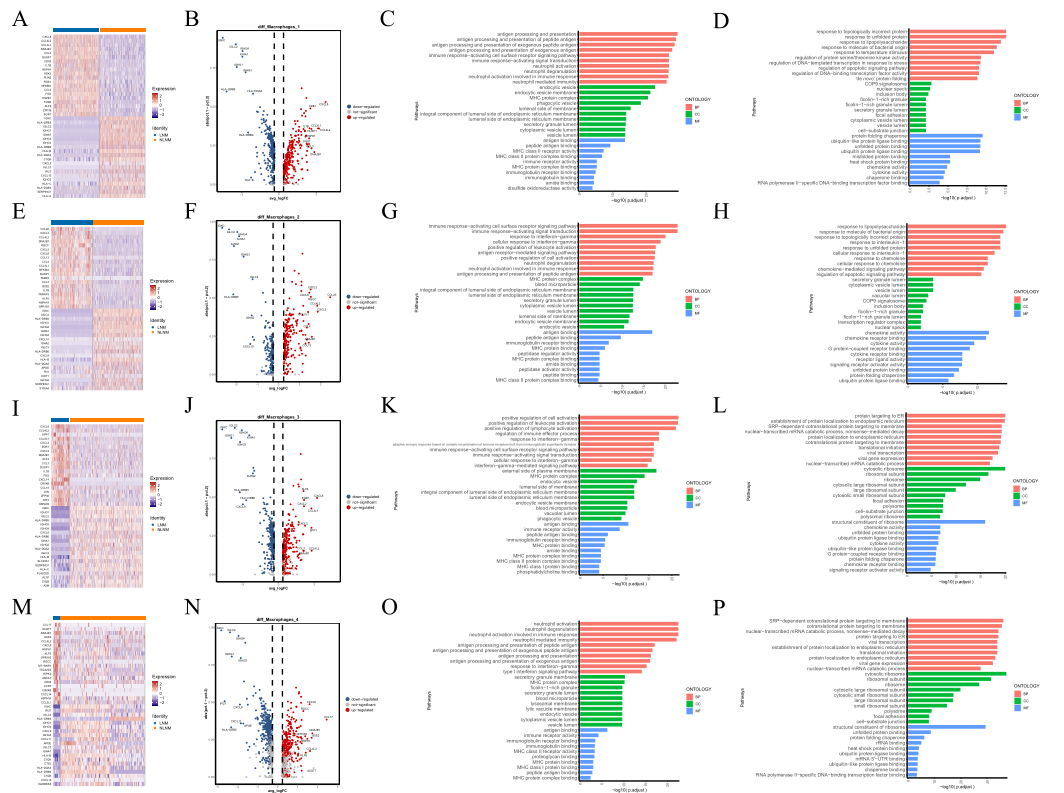
**

(A)Heatmap showing the differential expressed genes of type 1 macrophage. (B)Volcano plot showing the differential expressed genes of type 1 macrophage. (C)Barplots showing the upregulated pathways of type 1 macrophage. (D)Barplots showing the downregulated pathways of type 1 macrophage. (E)Heatmap showing the differential expressed genes of type 2 macrophage. (F)Volcano plot showing the differential expressed genes of type 2 macrophage. (G)Barplots showing the upregulated pathways of type 2 macrophage. (H)Barplots showing the downregulated pathways of type 2 macrophage. (I)Heatmap showing the differential expressed genes of type 3 macrophage. (J)Volcano plot showing the differential expressed genes of type 3 macrophage. (K)Barplots showing the upregulated pathways of type 3 macrophage. (L)Barplots showing the downregulated pathways of type 3 macrophage. (M)Heatmap showing the differential expressed genes of type 4 macrophage. (N)Volcano plot showing the differential expressed genes of type 4 macrophage. (O)Barplots showing the upregulated pathways of type 4 macrophage. (P) Barplots showing the downregulated pathways of type 4 macrophage (LNM: lymph node metastases; NLNM: non-lymph node metastases)

**Supplymentary figure 5. Biomarkers of macrophage clusters and hotspot module analysis.**

**
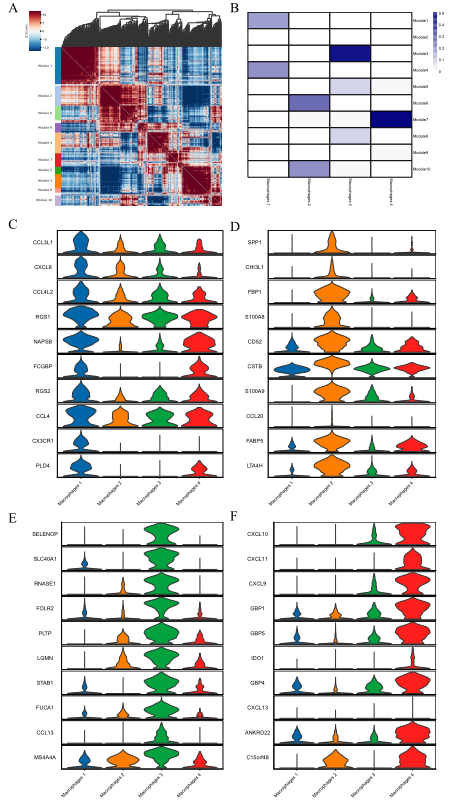
**

(A)Heatmap of macrophage genes modules information. (B) Relationship of macrophage genes module
